# IP-10 and CXCR3 signaling inhibit Zika virus replication in human prostate cells

**DOI:** 10.1371/journal.pone.0244587

**Published:** 2020-12-30

**Authors:** Jennifer L. Spencer Clinton, Linda L. Tran, Megan B. Vogt, David R. Rowley, Jason T. Kimata, Rebecca Rico-Hesse

**Affiliations:** 1 Department of Molecular Virology and Microbiology, Baylor College of Medicine, Houston, Texas, United States of America; 2 Integrative Molecular and Biomedical Sciences Graduate Program, Baylor College of Medicine, Houston, Texas, United States of America; 3 Department of Molecular and Cellular Biology, Baylor College of Medicine, Houston, Texas, United States of America; Faculty of Science, Ain Shams University (ASU), EGYPT

## Abstract

Our previous studies have shown that Zika virus (ZIKV) replicates in human prostate cells, suggesting that the prostate may serve as a long-term reservoir for virus transmission. Here, we demonstrated that the innate immune responses generated to three distinct ZIKV strains (all isolated from human serum) were significantly different and dependent on their passage history (in mosquito, monkey, or human cells). In addition, some of these phenotypic differences were reduced by a single additional cell culture passage, suggesting that viruses that have been passaged more than 3 times from the patient sample will no longer reflect natural phenotypes. Two of the ZIKV strains analyzed induced high levels of the IP-10 chemokine and IFNγ in human prostate epithelial and stromal mesenchymal stem cells. To further understand the importance of these innate responses on ZIKV replication, we measured the effects of IP-10 and its downstream receptor, CXCR3, on RNA and virus production in prostate cells. Treatment with IP-10, CXCR3 agonist, or CXCR3 antagonist significantly altered ZIKV viral gene expression, depending on their passage in cells of relevant hosts (mosquito or human). We detected differences in gene expression of two primary CXCR3 isoforms (CXCR3-A and CXCR3-B) on the two cell types, possibly explaining differences in viral output. Lastly, we examined the effects of IP-10, agonist, or antagonist on cell death and proliferation under physiologically relevant infection rates, and detected no significant differences. Although we did not measure protein expression directly, our results indicate that CXCR3 signaling may be a target for therapeutics, to ultimately stop sexual transmission of this virus.

## Introduction

Zika virus (ZIKV) is a single-stranded RNA *Flavivirus* that has gained attention in recent years for its ability to cause neurological impairments and birth defects [1–3]. While typically transmitted by *Aedes* species mosquitoes, ZIKV has also been classified as a sexually-transmitted infection [4–7]. ZIKV-infected males can secrete viral RNA and infectious virus in their semen up to 370 and 69 days, respectively [8–10], with the infective period of semen to others (female or male recipients) yet to be determined. This long-term viral secretion indicates ZIKV may establish persistent infections to develop in the urogenital tract. It is hypothesized that other viruses, such as Human Immunodeficiency Virus (HIV), Hepatitis C virus (HCV), cytomegalovirus, and human papillomavirus, are able to develop persistent infections by altering specific cytokine responses responsible for increasing host cell susceptibility or augmenting viral replication [11–14]. However, little is known about which persistence mechanisms mediate long-term ZIKV sexual transmission. Sexual transmission of ZIKV has been a focus of our research, which aims to elucidate factors related to urogenital tract tropism and transmission [15].

Primary innate immune responses elicit an initial anti-viral response that can dampen viral pathogenicity. Expression of interferons (IFNs) are a well-studied innate immune mechanism, eliciting downstream expression of IFN-stimulated genes (ISGs) [16]. Although ISGs are classically known to encode proteins with anti-viral effector functions, some ISGs have demonstrated pro-viral activity across *in vivo* and *in vitro* models of sexually-transmitted viral infections [17]. While ZIKV has been shown to elicit an anti-IFN response during infection, specific IFNs and ISGs have demonstrated the ability to decrease ZIKV infection in human cervical cancer cells, lung epithelial cells, and placental trophoblasts [18, 19]. However, the IFN response has yet to be characterized in the context of human prostate infection and ZIKV sexual transmission.

Furthermore, it has been shown that passage history and producer cell types of viruses can alter their infectivity profiles [20–23]. Isolates of ZIKV with high culture passage number do not recapitulate native infection as well as low-passage isolates, because they have been adapted to grow in cell culture, and they produce plaques [24–26]. Another caveat to using higher passage viruses during pathogenesis studies is that most of them are passaged through multiple cell types, or cell types that are not relevant to natural infections (i.e. Vero cells) [20, 26]. Thus, passaging through different cell types imposes various selective pressures on the virus, resulting in mutations that may cause altered infectivity. For example, mammalian and mosquito cell types express variable glycosyltransferases and glycosidases that cause altered glycosylation profiles on mature virions [27]. As ZIKV virions undergo maturation through the Golgi, the envelope glycoproteins are processed with glycosylation moieties that alter binding affinities for cellular receptors [28, 29]. Thus, viruses replicating in mosquitoes, which are injected into the skin, contain high mannose sialic acid moieties, while those that ultimately would infect the urogenital tract would contain moieties derived from epithelial or immune system cells of the same host. This lectin switching has been shown to occur in numerous other mosquito-borne viruses, including dengue virus (DENV), Japanese encephalitis virus, and West Nile virus (WNV) [30–34].

Previously, we demonstrated that ZIKV infects human prostate stromal mesenchymal stem cells (MSCs) and immortalized prostate epithelial cells, elucidating the prostate as a tropic site for ZIKV urogenital tract replication [15]. Therefore, we wanted to assess IFN/ISG production using low-passage ZIKV strains, evaluate potential anti-viral effects during ZIKV prostate cell infection, and delineate differences elicited by changes in propagating cell type or passage level. We have had the unique opportunity to analyze the phenotypes of three ZIKV strains, since we isolated them directly from patient serum and performed next generation sequencing of these isolates after passage one [35, 36]. In this paper, we show that genetically similar ZIKV isolates have unique growth phenotypes, cytokine profiles, and responses to anti-viral IFN-mediated signaling. We also describe a novel, anti-ZIKV role for a downstream ISG chemokine signaling pathway, CXCR3.

## Materials and methods

### Cells

Cells used in these experiments include 19I prostate stromal MSCs, LNCaP prostate epithelial adenocarcinoma cells (ATCC #CRL-1740), and PNT1A normal prostate epithelial cells (Sigma Aldrich #95012614). 19I cells are stromal MSCs derived from a healthy prostate donor (provided by D. Rowley), and maintain normal phenotypes when passaged in culture for several months [37]. They are non-transformed stem cells, can be differentiated into most mesenchymal cell lineages, have not been genetically-engineered, are phenotypically identical in surface markers to primary bone marrow-derived MSCs, and behave similarly as bone-marrow derived MSCs in culture [37]. These stem cells maintain some innate prostate-specific characteristics, but all factors of the prostate stem cell niche (tissue micro-environment and hormonal regulators) are not present in this system. 19I cells were maintained in Bfs media composed of high glucose DMEM (Gibco) supplemented with 5% (v/v) FBS (HyClone), 5% (v/v) NuSerum (Collaborative Research), 0.5 μg/mL testosterone (Sigma), 5 μg/mL insulin (Sigma), 100 units/mL penicillin (Sigma), and 100 μg/mL streptomycin (Sigma) at 37°C with 5% CO_2_. LNCaP cells were maintained in high glucose RPMI-1640 with L-glutamine and HEPES (ATCC), supplemented with 10% (v/v) FBS (Atlanta Biologicals), 100 units/mL penicillin, and 100 μg/mL streptomycin. PNT1A cells were maintained in high glucose RPMI-1640 with L-glutamine and HEPES (Gibco), supplemented with 10% (v/v) FBS (Atlanta Biologicals), 100 units/mL penicillin, and 100 μg/mL streptomycin.

### Virus strains

**Fig 1:**
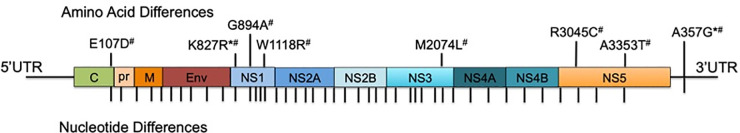
Genomic differences in ZIKV clinical isolates. Pictured here is the genome comparison of ZIKV strains FLR, FLA, and HN16 using ZIKV FLR as the reference. Each amino acid difference is noted in the polyprotein and those viruses containing the change are noted as follows: * = ZIKV FLA and # = ZIKV HN16. There is one nucleotide difference in the 3’UTR that is found in both FLA and HN16. Each nucleotide difference is denoted with a tick mark.

Three ZIKV clinical isolates were used in these experiments. ZIKV-FLR (GenBank: KU820897.5, passage 1) was isolated in *Ae*. *albopictus* C6/36 cells, from serum of an individual who travelled to Colombia in December 2015 [35]. FLR/1 was passaged twice in C6/36 cells prior to infections, while FLR/2 was passaged once in C6/36 cells and once in monocyte-derived dendritic cells (moDCs) prior to infections. Both FLR/1 and FLR/2 used for all studies were passage 2. ZIKV-FLA (GenBank: KY989971, passage 1) was isolated in primary human moDCs, from serum of a viremic subject in Florida, who was also infected in Colombia during December 2015. We were unable to isolate initial ZIKV-FLA from serum in C6/36 or Vero cells, and therefore it was isolated and propagated in primary human moDCs. FLA/1 was passaged three times in primary human moDCs prior to infections, while FLA/2 was passaged twice in moDCs and once in C6/36 cells prior to infections. Both FLA/1 and FLA/2 used for all studies were passage 3. All primary human moDCs used for virus passaging were prepared from anonymous donor blood. ZIKV-HN16 (GenBank: KY328289, passage 1), was isolated in Vero cells, from a viremic subject in Houston, Texas who had recently traveled to Honduras [36]. These ZIKV strains differ by 14–41 nucleotides by pairwise comparison, and code for 7 amino acid differences in total (**Fig 1**). There are no nucleotide differences in the 5’ UTR and one nucleotide difference within the 3’ UTR. All passage two ZIKV isolates were also deep sequenced, and resulted in no amino acid differences from passage one isolates. In studies done by others, ZIKV-FLR was passaged up to eight times in Vero or C6/36 cells, and resulted in 99.999% nucleotide sequence identity across 26 different FLR-derived isolates [38]. Furthermore, all three of the ZIKV isolates used in this study replicate similarly in C6/36 mosquito cells, commonly used for flavivirus propagation (**S1 Fig**). Vero (monkey kidney) cells were also compared as a common passaging cell type, and showed similar growth kinetics between ZIKV isolates FLR and HN16, with significantly higher replication seen with FLA. Dengue virus serotype 2 (DENV-2) strain K0049, a non-sexually transmitted *Flavivirus*, was propagated three times in mosquito cells, from patient serum, and was used for comparison [39]. Most flavivirus clinical isolates do not form plaques after very low passage *in vitro*; others have passaged ZIKV strains to develop plaques but we believe this is due to artificial selection (e.g. Puerto Rico, French Polynesia strains). Therefore, we have shown infectivity of these virus isolates by limiting dilution assays instead.

### Viral infections

19I, LNCaP and PNT1A cells were infected with passage 2 or 3 of ZIKV strains FLA, FLR or HN16 at an multiplicity of infection (MOI) of 1 or 0.1 for 2 hours at 37°C with 5% CO_2_. MOI was calculated based on the RNA copies/mL and TCID/mL of each virus and the number of cells to be infected. Virus was removed and cells were washed with 1× PBS (HyClone) three times and new media replaced. Infected cells were cultured in RPMI-1640 supplemented with 2% (v/v) FBS and no antibiotics (LNCaP and PNT1A), or Bfs media (19I stromal MSCs). Cells were kept at 37°C with 5% CO_2_ up to 7 days post-infection (dpi) and 50 μl of supernatants collected daily in TRIzol LS (ThermoFisher), and stored at -70°C. 50 μl of fresh media was replaced daily to ensure consistent volume. UV-inactivated ZIKV-FLR was used as a negative control and cells were infected with equivalent RNA copies. FLR was exposed to 45 joules/cm^2^ of UV irradiation with a Stratagene UV crosslinker.

19I stromal MSCs or PNT1a cells were treated with 3.75 ng/mL recombinant human CXCL10 (IP-10) protein (Tonbo) 3 hours before infection, or 24 hours after infection, at 37°C with 5% CO_2_. After pre-treatments, all media containing IP-10 was removed prior to ZIKV infection. In separate experiments, prostate cells were treated with 3.75 ng/mL CXCR3 agonist, PS372424 (Millipore Sigma), or CXCR3 antagonist, (±)-NBI 74330 (Tocris), 3 hours prior to ZIKV infection. All media containing PS372424 or (±)-NBI 74330 was removed prior to ZIKV infection.

### Limiting dilutions assays

Limiting dilutions were used to determine infectious particles in critical assays, to demonstrate that viral RNA levels do correspond to infectious virion production, because we cannot perform plaque assays (these viruses do not produce plaques). Dilution assays were performed as previously described [15] with the following changes. Briefly, Vero cells (clone E6; CRL-1568, ATCC) were seeded onto 48 well plates 24 hours before experiments. Cell supernatants were collected from ZIKV-infected PNT1a cells 5 days after infection and serially diluted using 10-fold dilutions (up to 10^8^). Diluted cell supernatants were added directly to Vero cells and incubated for 1 hour at 37°C with 5% CO_2_. Inoculum was replaced with fresh medium and kept at 37°C with 5% CO_2_. Five days after infection, Vero cells were fixed with cold acetone and methanol (1:1), and non-specific staining blocked with TBS SuperBlock (ThermoFisher). Cells were stained by immunofluorescence for flavivirus envelope protein using the primary mouse 4G2 monoclonal antibody (EMD Millipore; 1:50), FITC anti-mouse secondary antibody (Cat# M32201, ThermoFisher; 1:100), and NucBlue DAPI nuclear stain (ThermoFisher). Vero cell monolayers were observed for immunofluorescent staining by fluorescent microscopy and scored as a positive well if any fluorescent cells were observed. In tandem, viral RNA copy number of the initial supernatants were quantified by qRT-PCR (see Quantitative RT-PCR section). Comparison of the lowest infectious dilution with the number of viral RNA copies detected at the time of supernatant collection allowed us to calculate tissue culture infective dose as a representation of the infectious virus titer.

### Multiplex cytokine bead array assay

Cytokine and chemokine levels in cellular supernatants were determined using the Milliplex 41-plex human cytokine/chemokine magnetic bead panel (Millipore) according to the manufacturer’s instructions. Samples were collected on either the Bio-Plex (Bio-Rad) using Bio-Plex Manager software or the Magpix (Millipore) using xPonent software (Luminex). Cytokines analyzed are listed in **S1 Table**. Heat maps and Principal Component Analyses (PCAs) were generated using Orange3 software (version 3.27.1).

### Quantitative RT-PCR

Viral RNA was extracted from supernatants collected from prostate cells infected with ZIKV or DENV-2 using TRIzol LS, according to manufacturer's instructions. Quantitative RT-PCR (qRT-PCR) was performed using primers and probes for the ZIKV envelope gene or DENV-2 capsid gene [40–42] using TaqMan Fast Virus 1-Step Master Mix kit (Applied Biosystems), according to manufacturer's instructions. Concentrations of viral RNA (copies/mL) were determined by comparison to a standard curve generated from ZIKV-FLR or DENV-2 transcripts, run simultaneously. Sequences for ZIKV or DENV-2 primers and probes are described in **S2 Table.**

Due to a lack of isoform-specific antibodies, abundance of CXCR3 isoforms can only be measured by qRT-PCR. Whole prostate cell RNA was collected by lysing cells with TRIzol (ThermoFisher) according to manufacturer’s instructions. Cellular RNA was extracted using Direct-zol RNA Miniprep Kit (Zymo) and quantified using a Zone 3 spectrophotometer (Epoch). Quantitative RT-PCR was performed using primers for the CXCR3 chemokine receptor isoforms [43] using the Power SYBR Green RNA-to-C_T_ 1-step kit (Applied Biosystems). Levels of generic CXCR3 are not directly proportional to levels of CXCR3-A and CXCR3-B isoforms, due to differences in isoform lengths and qRT-PCR primer recognition sites. GAPDH was used as an endogenous control for relative comparisons. All reactions were performed on a StepOnePlus Real-Time PCR system (Applied Biosystems). Fold change in CXCR3 expression was reported as 2^-ΔΔC^_T_.

### Immunofluorescence

Immunofluorescence assays were performed by fixing ZIKV-infected cells to black-walled, optical bottom 96 well plates (ThermoFisher) with Cytofix/Cytoperm Solution (BD) for 20 minutes and washed with 1× Perm/Wash buffer (BD). Cells were blocked with normal mouse serum (1:10, Sigma) for 10 minutes at room temperature, and incubated with rabbit CXCR3 IgG monoclonal antibody (mAb) (1:250, ThermoFisher, 6H1L8) for detection of membrane bound and cytosolic CXCR3 for 1 hour at 37°C. Cells were washed with 1× Perm/Wash buffer and incubated with AlexaFluor594 goat anti-rabbit IgG secondary antibody (1:500, Invitrogen, A11012) at 37°C for 1 hour. Cells were washed with Perm/Wash buffer, stained with NucBlue reagent (Invitrogen) and stored in 1× PBS at 4°C prior to imaging. To detect only membrane bound CXCR3, the same procedure was used with Cytofix reagent (BD) for fixation and 1× PBS with 2% FBS for washing. Imaging was performed on a Nikon A1-R Confocal Microscope.

### Cellular viability assays

ZIKV infected and uninfected prostate cells were removed from wells and assessed for cellular viability at 7 dpi. Cells were fixed and permeabilized with Cytofix/Cytoperm Solution, and stained with Ghost-Dye 780 (Tonbo) for 30 minutes at 4°C in the dark. Cells were washed twice with 1× PBS with 2% FBS, and assessed for viability by flow cytometry using a BD FACSCantoII Cytometer and FACSDiva software (v8.0.3). Flow cytometry data was analyzed using FlowJo software (version 10.2).

### Cellular proliferation assays

Prostate cells were stained with 1μM CellTrace Violet Cell Proliferation Kit (Thermo Fisher) according to manufacturer’s instructions. Briefly, cells were split one day before experimentation to ensure cellular division. Cells were serum-starved for approximately 24 hours prior to staining. Cells were stained for 20 minutes with 1μM CellTrace Violet at 37°C + 5% CO_2_, and washed twice with complete media. Stained cells were then plated and infected as previously described. Cells were transferred to a 96 well plate using dissociation buffer (Millipore) and fixed with Cytofix/Cytoperm as previously described. Flow cytometry for cellular proliferation was performed at 7 dpi using the 96 well plate reader on the NXT Attune Acoustic Focusing Cytometer.

### Caspase activation assay

Activity of caspases 3 and 7 were measured by CaspaseGlo 3/7 activation assay (Promega), according to the manufacturer’s instructions. Briefly, prostate cells were plated in 96 well, white-walled, optical bottom plates (ThermoFisher). After 24 hours, cells were pre-treated with IP-10, CXCR3 agonist, or CXCR3 antagonist, and infected with ZIKV as previously described in the Viral Infections section. Approximately 24 hours post-infection, CaspaseGlo reagent was added to wells in a 1:1 ratio, shaken for 30 seconds at 300 rpm, and incubated for 1 hour at room temperature. Positive controls were PNT1a and stromal MSCs incubated at 55°C or 60°C to induce heat shock and caspase activation. Negative background controls were media alone with no cells. Luminescence was recorded on a Spectromax5 Plate Reader (Molecular Devices). Reported relative light units (RLUs) are the averages of 5 points within individual wells.

### Statistical analysis

All statistical analyses were performed using GraphPad Prism software (version 6). Data were assessed for normality using the D'Agostino & Pearson omnibus normality test. Standard error of the mean (SEM) was used as a measure of variance for all data sets. If normally distributed, data were analyzed by repeated measures two-way ANOVA with multiple comparisons t-tests using Tukey correction. If not normally distributed, data were analyzed by Friedman’s Test with Dunn’s multiple comparisons and used to determine significant differences between three or more groups. A two-tailed Mann-Whitney test was used to determine significant differences between two groups. *P*-values <0.05 were used to denote significance. All experiments were performed in either duplicate or triplicate biological replicates, with two or three technical replicates each.

## Results

### ZIKV and DENV-2 isolates elicit distinct cytokine profiles in prostate cell infections

**Fig 2:**
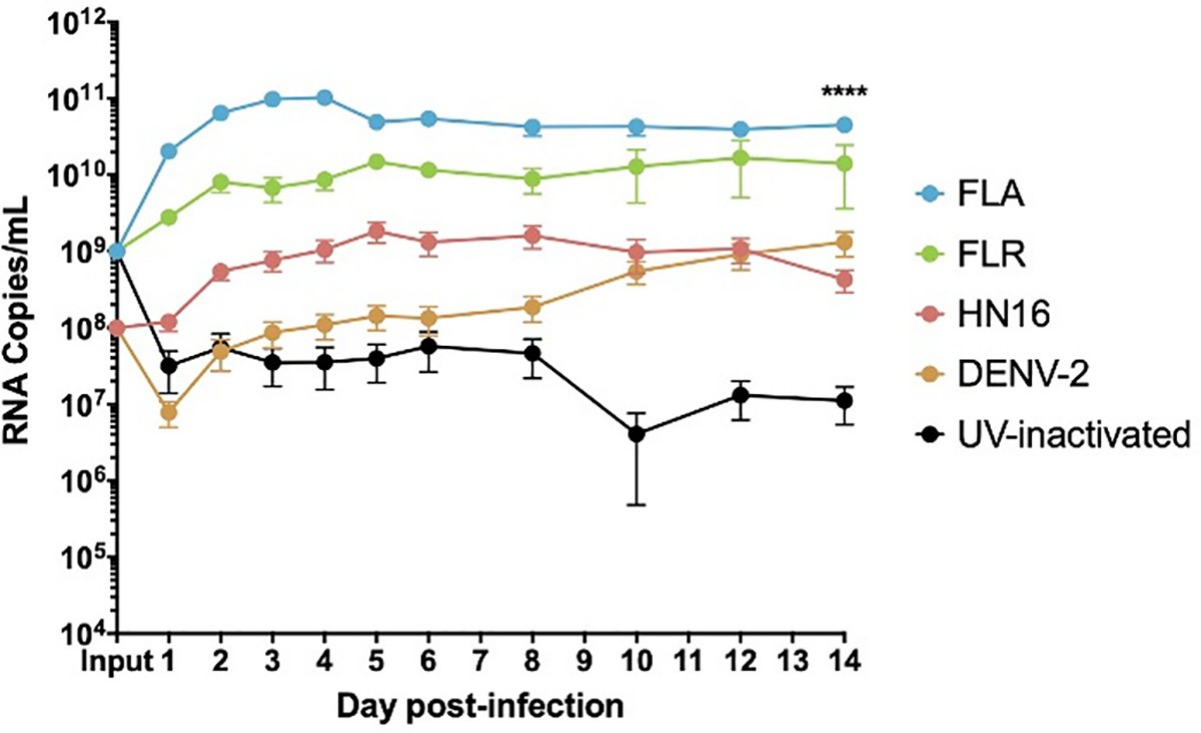
ZIKV isolates have distinct growth phenotypes in prostate epithelial cells. Infected epithelial cell supernatants were collected and RNA extracted daily up to 14 dpi. One-step qRT-PCR was performed with ZIKV specific primer and probe to assess viral replication. Growth curves of ZIKV isolates FLA, FLR, HN16, DENV-2, and UV-inactivated FLR were measured in PNT1a cells at an MOI of 1. Data are from 2 or 3 independent experiments with 3 technical replicates each. Error bars are SEM. Statistical significance was determined using the D'Agostino & Pearson omnibus normality test, and a Friedman Test with Dunn’s multiple comparisons. Significance was **** = *p*<0.001, comparing differences between all isolates over the 14-day time course.

Our initial report of ZIKV infection in prostate cells showed infectivity differences for virus isolates in prostate stromal MSCs and LNCaP epithelial cells [15]. The LNCaP line has an epigenetically silenced STAT2 gene and cannot fully signal through the IFNα/β/λ pathways [44]. Therefore, here we used the PNT1a STAT2-producing prostate epithelial cell line to study the full breadth of IFN signaling pathways in the prostate epithelium. Prior to assessing IFN/ISG production, ZIKV replication in PNT1a epithelial cells was evaluated by infecting cells at an MOI of 1, and quantifying viral RNA by qRT-PCR. All three ZIKV isolates replicated at significantly different levels in PNT1a cells (**Fig 2**). UV-inactivated ZIKV RNA persisted in the culture media at significantly lower levels out to 14 dpi, as was also evident in our previous studies utilizing the same inactivated virus stocks [15]. Previous studies by our group have shown that DENV RNA is able to persist in media, even in cell-free environments [45]. These data indicate UV-inactivated virions can persist in culture media after adequate washing, without actively replicating. Therefore, these data indicated suitability for studying cytokine effects on differential replication in the PNT1a human prostate epithelial line.

To probe the role of IFNs in ZIKV differential RNA and virion replication, we measured induction of 41 cytokines during ZIKV or DENV-2 infection of prostate stromal MSCs, LNCaP epithelial, and PNT1a epithelial cells by multiplexed immunoassay. Our results show that all three ZIKV isolates and DENV-2 elicit distinct cytokine profiles in infected prostate epithelial cells and stromal MSCs (**S2–S4 Figs**). LNCaP epithelial cells, PNT1a epithelial cells, and stromal MSCs elicited differential induction of cytokines based on ZIKV isolate or DENV-2 infection, as shown by heat maps (**S2–S4 Figs**). PCA was used to assess correlations between specific cytokines and viral infection based on isolate. Each ZIKV isolate and DENV-2 elicited distinct cytokine profiles during infection of prostate epithelial cells or stromal MSCs, as demonstrated by clustering of viral isolates in PCA (**S2–S4 Figs**). The top cytokines contributing to principal component clustering in LNCaP ZIKV infections included growth factors and pro-inflammatory cytokines (**Table 1**). As expected, LNCaP epithelial cells demonstrated no contribution of IFN/ISGs to overall clustering of isolates. Stromal MSCs and PNT1a epithelial cells showed an increase in IFNs (IFNα2 or IFNγ, respectively) and IFN-related proteins, contributing to the clustering of the ZIKV isolate cytokine profiles compared to DENV-2 (**S2–S4 Figs**). Furthermore, DENV-2 induced diverse cytokine profiles in each prostate cell type investigated, and yielded cytokine production similar to that of ZIKV-HN16. Taken together, these data indicated that ZIKV and DENV-2 infection of prostate cells induced distinct cytokines during infection based on the isolate. Furthermore, we demonstrated that IFNs/ISGs induced during ZIKV or DENV-2 infection of PNT1a epithelial cells and stromal MSCs contributed to the distinct clustering of isolates.

**Table 1:** Cytokines most contributing to distinct clustering of each ZIKV isolate and prostate cell type.

PNT1a		LNCaP		MSC	
PC1	PC5	PC1	PC4	PC1	PC4
TNFα	FGF-2	GM-CSF	RANTES	IFNα2	MIP-1α
IL-12 (p70)	IFNγ	IL-4	IL-9	IL-1β	IP-10
IL-9	EGF	MDC	MIP-1β	IL-13	RANTES
PDGF-BB	sCD40L	MCP-3	TNFα	IL-12 (p40)	FGF-2

The top five cytokines in each principal component analysis contributing to clustering of specific ZIKV isolates are listed for PNT1a, LNCaP, and MSC prostate cells. Most highly contributing cytokines were derived from PCAs, including all time points in the study.

### IFNγ and IP-10 are significantly upregulated in ZIKV infection of prostate cells

**Fig 3:**
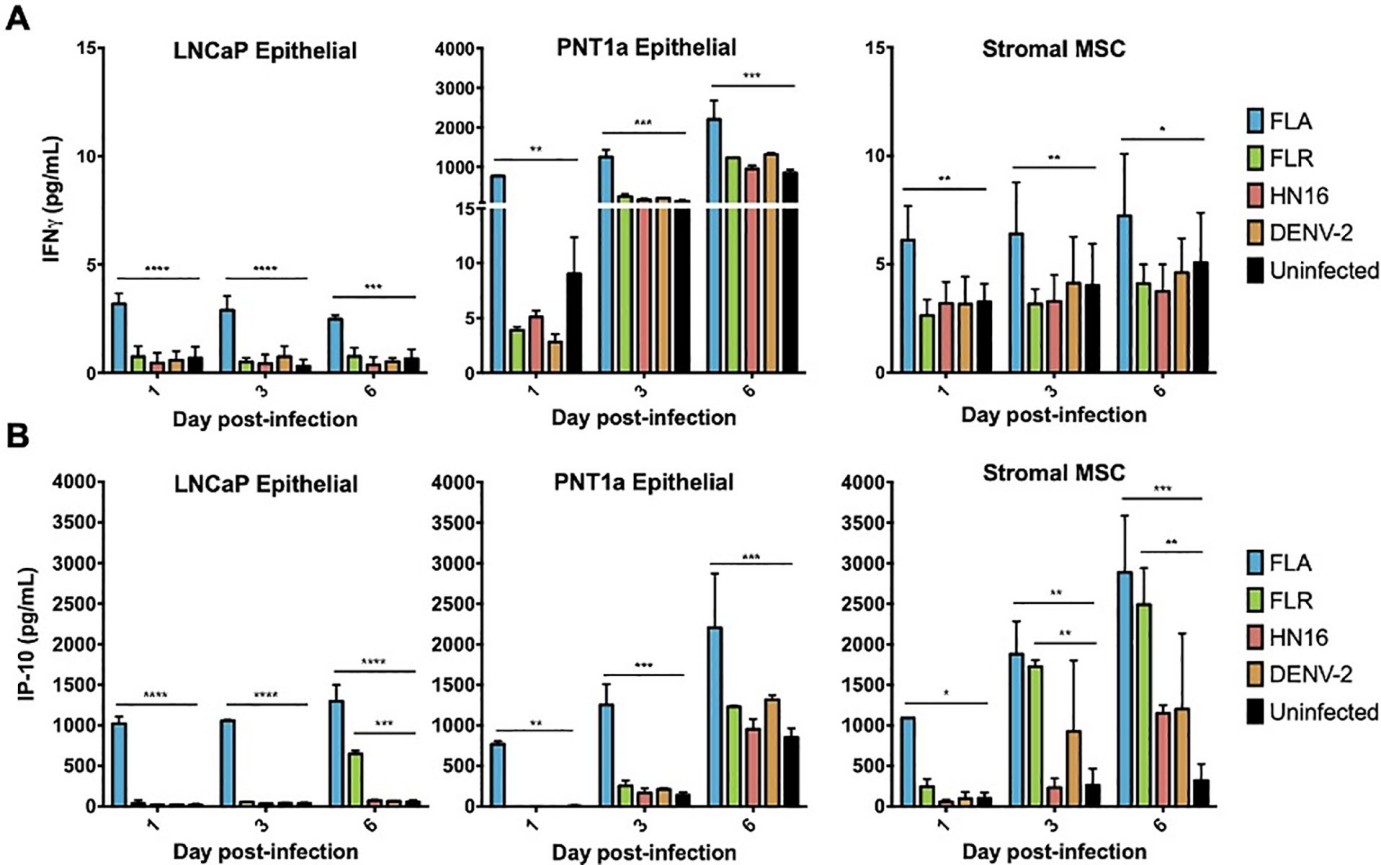
IFNγ and IP-10 are significantly upregulated during ZIKV infections of three different prostate cell types. Infected cell supernatants were collected at 1, 3, and 6 dpi and assessed for expression of cytokines using MAGPIX multiplex immunoassay. Expression of IFNγ (**A**) or IP-10 (**B**) in pg/mL from ZIKV FLA, FLR, HN16, or DENV-2 infection. Data are from 2 independent experiments with 3 technical replicates each. Error bars are SEM. Statistical significance was determined by repeated measures two-way ANOVA with Dunnett’s multiple comparison test. Significance was * = *p*<0.05; ** = *p*<0.01; *** = *p*<0.005; **** = *p*<0.001, comparing ZIKV isolates to uninfected controls.

Two cytokines that contributed substantially to the cytokine profile clustering in ZIKV infection of stromal MSCs and PNT1a cells were IFNγ and IP-10. Our results show that IFNγ is significantly upregulated during ZIKV-FLA infection of prostate epithelial cells and stromal MSCs, as compared to uninfected controls (**Fig 3A**). While IFNγ is canonically made by immune cells [16], our data show that multiple prostate cell types produce IFNγ upon ZIKV infection. Furthermore, the ISG IP-10 (CXCL10) was expressed at significantly higher levels during infection with ZIKV-FLA in PNT1a cells, and with ZIKV-FLA or FLR infection in stromal MSCs, over uninfected controls (**Fig 3B**). Interestingly, while IFNγ levels remain fairly steady, IP-10 levels increase over the course of infection. IP-10 expression in FLA-infected PNT1a cells, and FLA- or FLR-infected stromal MSCs markedly increases between 1 dpi and 6 dpi. These data suggest that ZIKV infection upregulates IP-10 during initial infection of prostate cells, and levels of IP-10 continue to increase during infection.

### IP-10 reduces ZIKV replication in prostate cells in a passage-dependent manner

**Fig 4:**
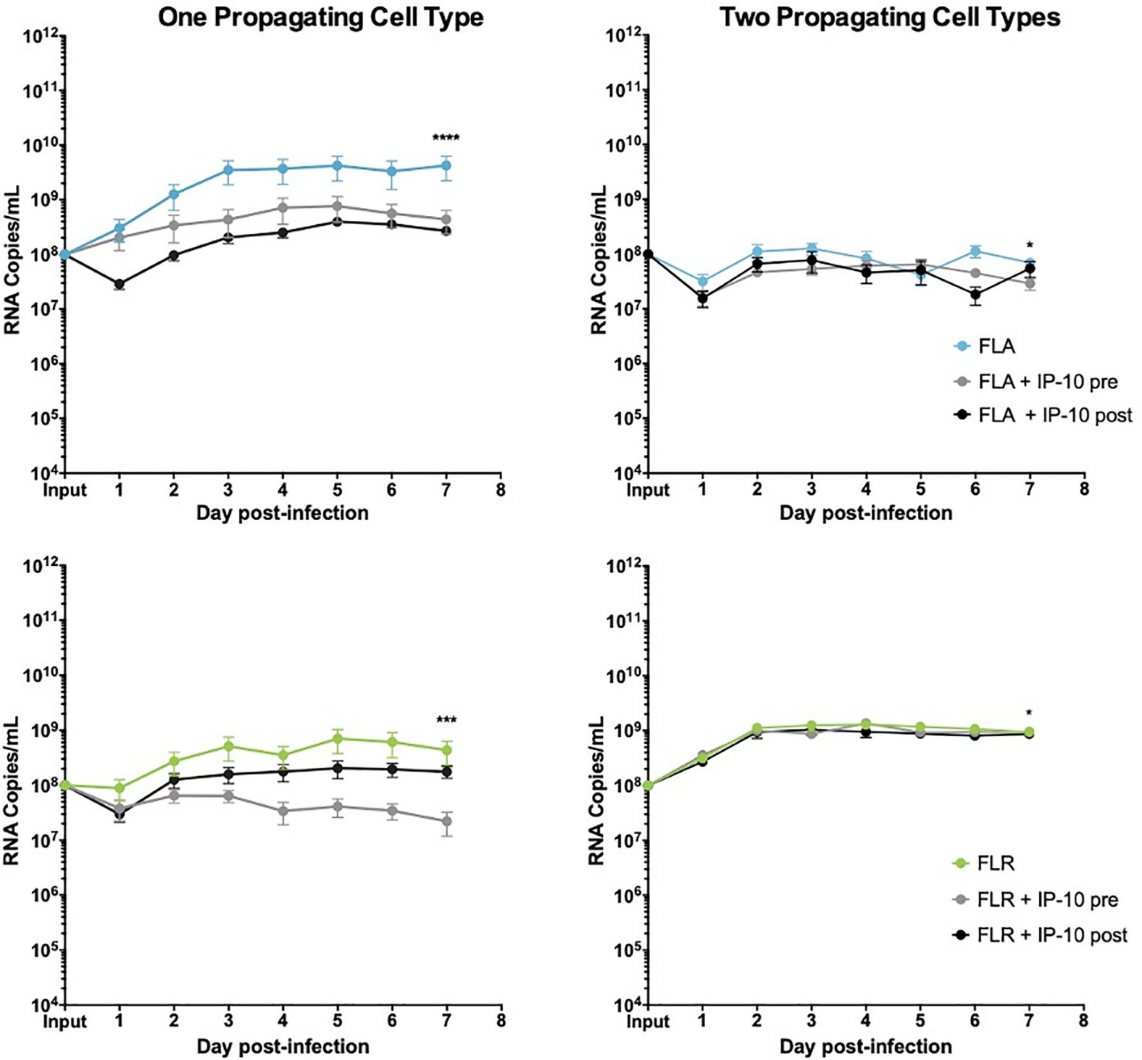
IP-10 treatment decreases ZIKV replication in low passage ZIKV infection of prostate epithelial cells. PNT1a epithelial cells were treated for 3 hours with 3.75 ng/mL human IP-10 prior to infection (pre), or 24 hours after infection (post). Cells were infected with ZIKV FLA or FLR propagated in only one cell type (left column), or after passage through two cell types (right column). Infected cell supernatants were collected and RNA extracted daily up to 7dpi. One-step qRT-PCR was performed with ZIKV specific primer and probe to assess viral replication. Data are from 2 or 3 independent experiments with 3 technical replicates each. Error bars are SEM. Statistical significance was determined using D'Agostino & Pearson omnibus normality test, and a repeated measures two-way ANOVA with multiple comparison t-tests using the Holm-Sidak correction. Significance was * = *p*<0.05; *** = *p*<0.005; **** = *p*<0.001, comparing differences in overall virus replication between treatment conditions.

To determine if IP-10 influences ZIKV replication, we treated prostate epithelial cells and stromal MSCs with 3.75 ng/mL recombinant human IP-10 three hours before, or 24 hours after infection with ZIKV-FLA or FLR at an MOI of 0.1. The amount of recombinant IP-10 used for treatments was based on the maximum amount of IP-10 produced during ZIKV-FLA infection of stromal MSCs (**Fig 3B**). ZIKV-HN16 was not included in these studies due to a lack of IP-10 induction during infection. Furthermore, we included ZIKV isolates propagated through one or two cell types to further demonstrate the effects of virus passaging on phenotypes. IP-10 treatment of PNT1a prostate epithelial cells before or after infection with ZIKV-FLA or FLR propagated through one cell type significantly reduced levels of ZIKV RNA compared to untreated infected cells by qRT-PCR (**Fig 4**). ZIKV-FLA propagated through a second cell type lost most replicative ability altogether and showed a slight decrease in viral RNA production when treated with IP-10. Conversely, ZIKV-FLR propagated through a second cell type replicated similarly to that of one propagating cell type. However, IP-10 treatment only had a slightly negative effect on ZIKV-FLR RNA production, specifically at 4 dpi.

Furthermore, because these low-passage isolates do not form plaques on monolayers, we used limiting dilution assays to determine changes in infectious ZIKV production (**Table 2**). Based on limiting dilutions, we showed that IP-10 treatment does not significantly alter the amount of infectious ZIKV produced during infection of PNT1a cells.

**Table 2:** Infectious ZIKV detected in infected cell supernatants, shown in TCID/mL.

ZIKV Isolates	PNT1a Cells		
	Untreated	IP-10	CXCR3 Agonist
Mock	ND	ND	ND
FLA/1 (one propagating cell type)	1×10^2^	1×10	1×10^2^
FLA/2 (two propagating cell types)	1×10	1×10	1×10
FLR/1 (one propagating cell type)	1×10	1×10	1×10
FLR/2 (two propagating cell types)	1×10^2^	1×10^2^	1×10^4^

Experiments were done in duplicate, showing highest level of ZIKV detected across both replicates. Infected supernatants were collected from PNT1a cells at 5 dpi, and used to infect Vero cell monolayers. Abbreviation: TCID, tissue culture infectious dose; ND, not detected.

**Fig 5:**
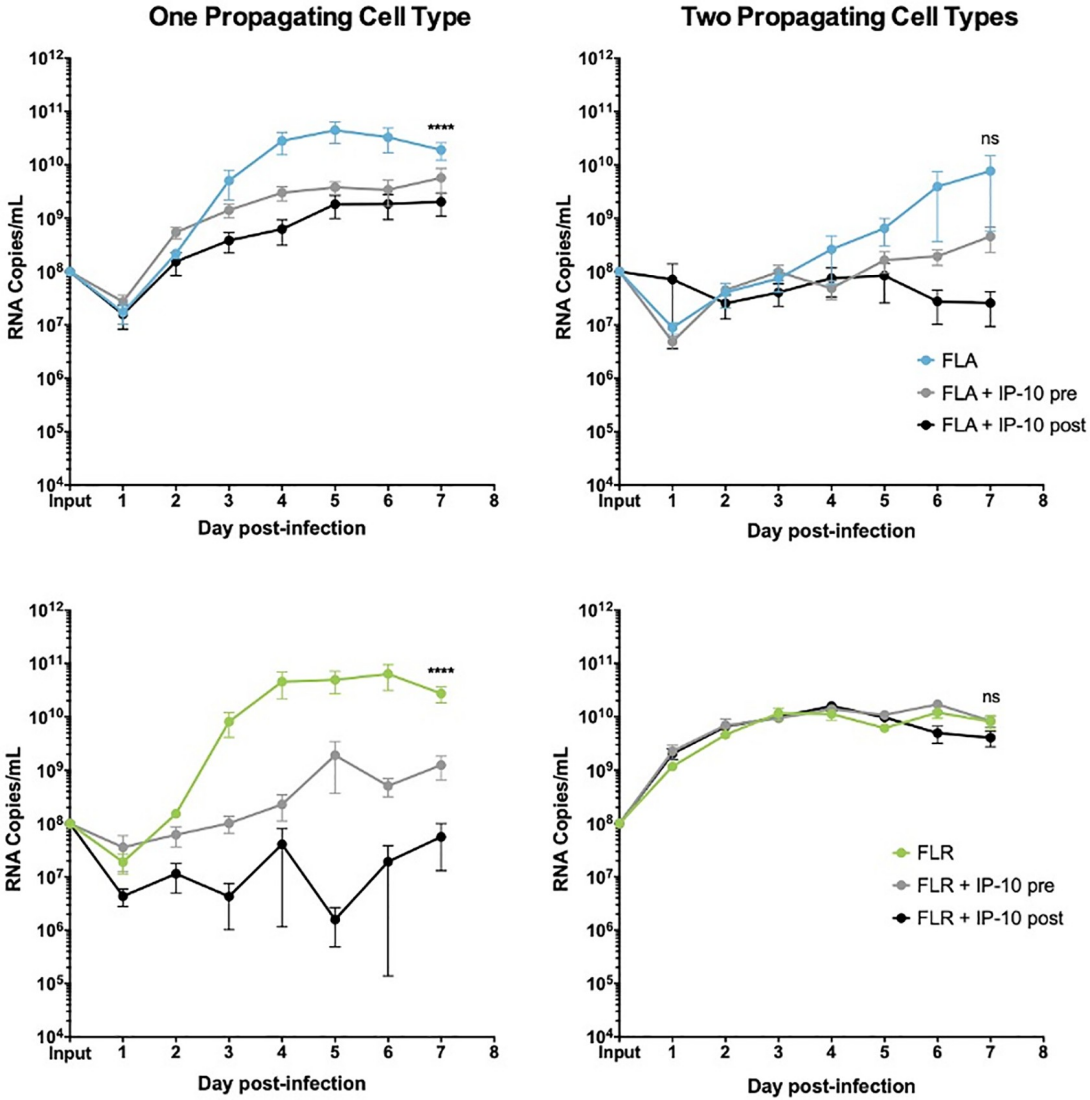
IP-10 treatment decreases ZIKV replication in low passage ZIKV infection of prostate stromal MSCs. MSCs were treated for 3 hours with 3.75 ng/mL human recombinant IP-10 prior to infection (pre), or 24 hours after infection (post). Cells were infected with ZIKV FLA or FLR propagated in only one cell type (left column), or after passage through two cell types (right column). Infected cell supernatants were collected and RNA extracted daily up to 7dpi. One-step qRT-PCR was performed with ZIKV specific primer and probe to assess viral replication. Data are from 2 or 3 independent experiments with 3 technical replicates each. Error bars are SEM. Normality was assessed using the D'Agostino & Pearson omnibus normality test. Statistical significance was determined using repeated measures two-way ANOVA followed by multiple comparison t-tests using the Tukey correction, or a Friedman Test with Dunn’s multiple comparisons test. Significance was ns = not significant; **** = *p*<0.001, comparing differences in overall virus replication between treatment conditions.

ZIKV replication and/or IP-10 anti-viral activity were diminished after propagating ZIKV-FLA or FLR through a second cell type as compared to propagation in one cell type. While IP-10 treatment inhibited replication in PNT1a epithelial cells, it only slowed viral replication kinetics in prostate stromal MSCs (**Fig 5**). Similar to epithelial cells, stromal MSCs lost IP-10 anti-viral activity when infected with ZIKV-FLA or FLR propagated through a second cell type. Based on these results, we concluded that IP-10 has an initial anti-viral effect during ZIKV infection of prostate cells, which is lost after propagation through multiple cell types.

### CXCR3 is upregulated during ZIKV prostate cell infection

**Fig 6:**
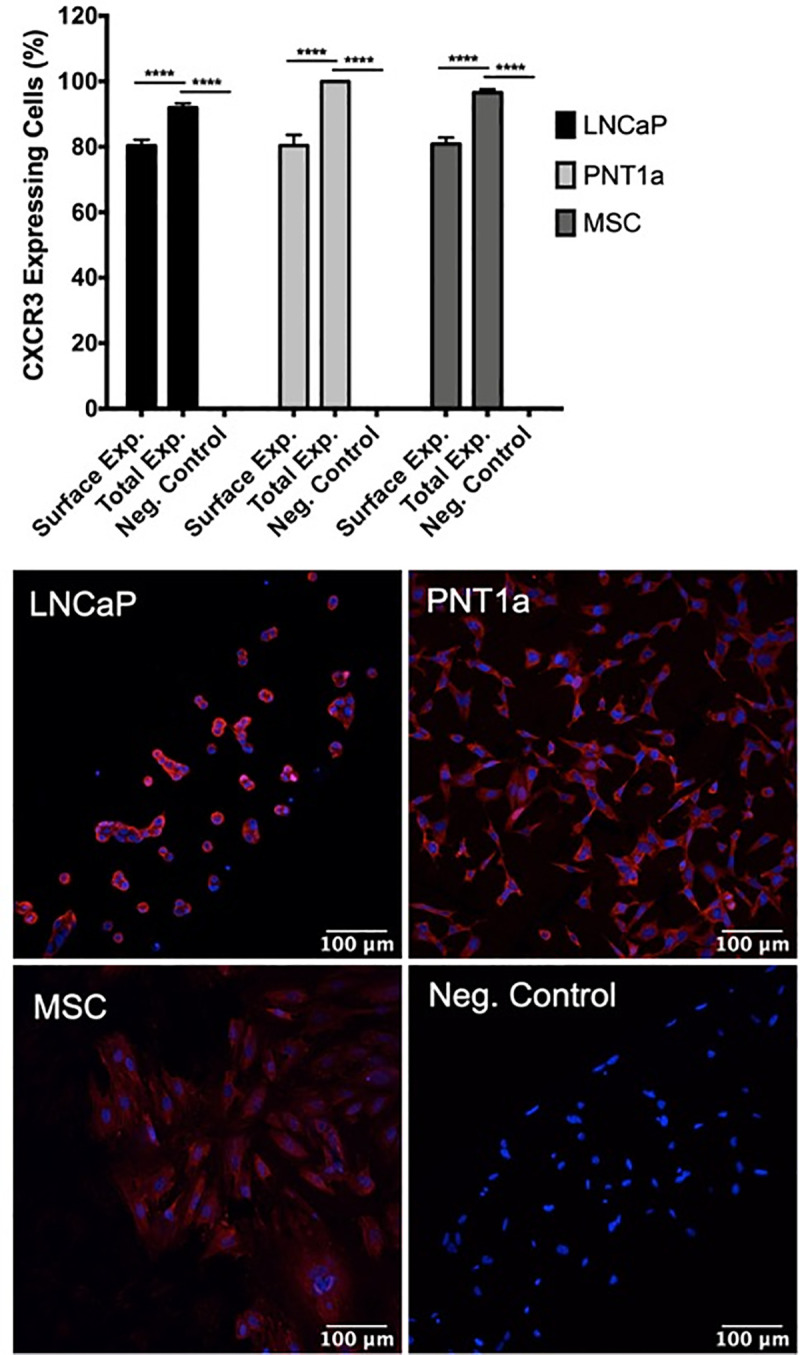
Prostate epithelial cells and mesenchymal stem cells express high levels of CXCR3. Uninfected cells were fixed, stained with CXCR3 specific antibody, and expression assessed by immunofluorescence. Surface expression and total expression of CXCR3 on prostate cells were quantified with ImageJ software. Negative controls consisted of cells stained without primary antibody. Data are from 2 independent experiments with 3 technical replicates each. Error bars are SEM. Statistical significance was determined using one-way ANOVA followed by multiple comparison t-tests using the Tukey correction. Significance was **** = *p*<0.001.

IP-10 signals through its cognate receptor, CXCR3, to control downstream effects on cellular proliferation and survival [46]. To assess CXCR3 protein expression on prostate cells, we stained epithelial and stromal MSCs with CXCR3-specific antibody and performed immunofluorescent imaging. Our results show that surface-expressed CXCR3 is expressed on approximately 80% of prostate PNT1a cells and prostate stromal MSCs, as compared to the LNCaP cell positive control and CXCR3 primary antibody unstained control cells (**Fig 6**). Differences in surface or total expression of CXCR3 were distinguished by treating cells with or without permeabilization solution during staining. Approximately 95–100% of PNT1a, stromal MSCs, and LNCaP cells express either surface-expressed or cytosolic CXCR3, and are expressed on a significantly higher percentage of cells than surface-expressed CXCR3 alone.

**Fig 7:**
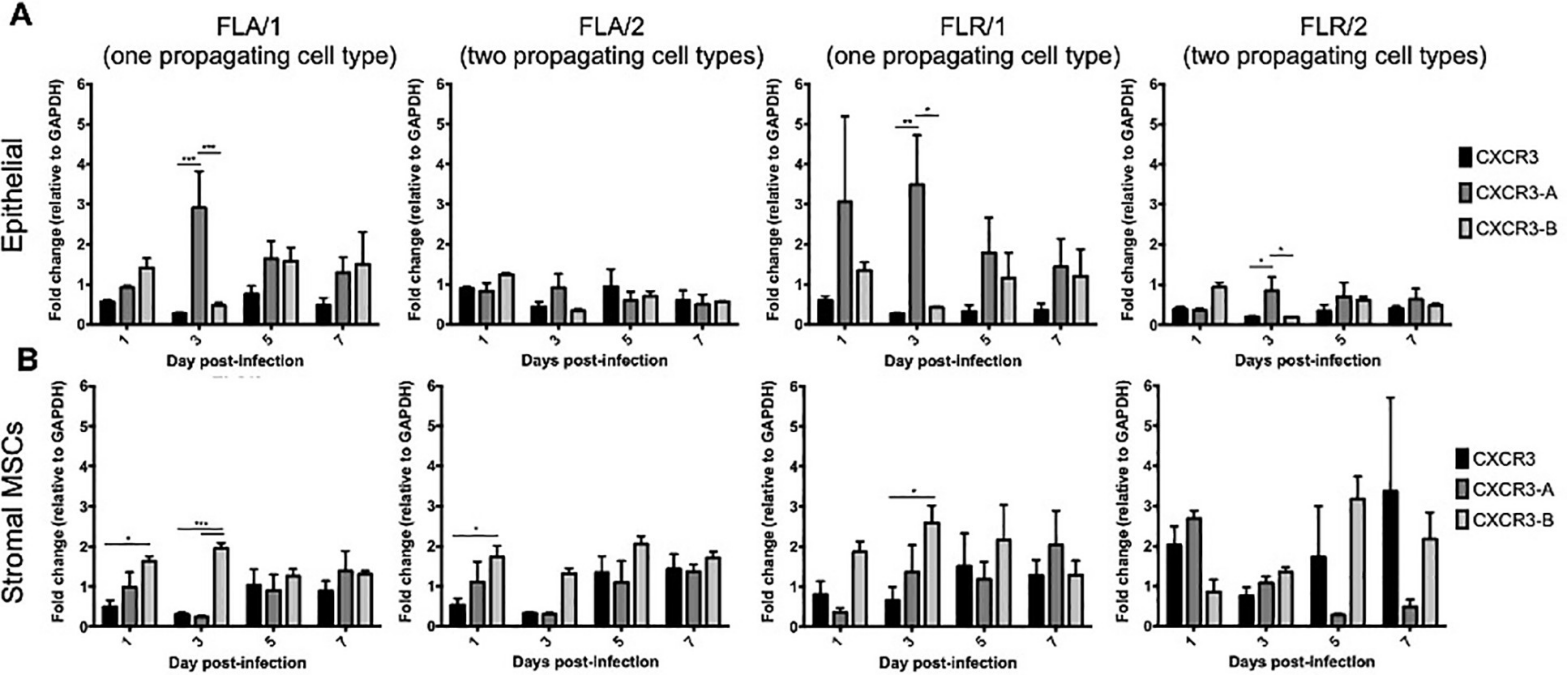
Prostate cells upregulate CXCR3 isoforms during ZIKV infection in a passage-dependent manner. Infected PNT1a (**A**) or MSCs (**B**) were collected, lysed, and total cellular RNA extracted at 1, 3, 5, and 7 dpi. One-step qRT-PCR was performed with generic CXCR3 primers or CXCR3 isoform-specific primers to assess gene expression. Data are represented as 2^-ΔΔCT^ fold change relative to uninfected cells and GAPDH. Number of cell types used to propagate virus are indicated numerically after ZIKV isolate names. Data are from 2 independent experiments with 2 technical replicates each. Error bars are SEM. Statistical significance was determined using two-way ANOVA followed by multiple comparison t-tests using the Tukey correction. Significance was * = *p*<0.05; ** = *p*<0.01; *** = *p*<0.005.

CXCR3 is expressed in two primary isoforms (CXCR3-A and CXCR3-B) that have alternative effects on proliferation and apoptosis induction [46, 47]. Signaling through isoform CXCR3-A leads to increased proliferation and decreased apoptosis, while signaling through CXCR3-B yields a decrease in proliferation and increase in apoptosis [46, 47]. To determine if CXCR3 is expressed on ZIKV-infected prostate cells, and which isoforms are present, we performed qRT-PCR for detecting transcribed RNA in prostate epithelial and stromal MSCs up to 7 dpi. Our results indicate that both isoforms are upregulated during ZIKV infection of prostate cells, as compared to GAPDH expression and uninfected cell controls (**Fig 7**). While both CXCR3 isoforms are upregulated during ZIKV infection, CXCR3-A is significantly more abundant on prostate epithelial cells during early infection with ZIKV-FLA or FLR propagated in one cell type (referred to as FLA/1 and FLR/1) (**Fig 7A**). CXCR3-A and CXCR3-B levels stabilized to similar levels during late ZIKV infection at 7dpi. However, ZIKV propagated through a second cell type (referred to as FLA/2 and FLR/2) had lower induction of both CXCR3 isoforms in epithelial cells, compared to that of ZIKV propagated from one cell type. Alternatively, ZIKV-FLA or FLR propagated through a single cell type significantly upregulated CXCR3-B expression in stromal MSCs during early steps of infection (**Fig 7B**). Prostate stromal MSCs infected with ZIKV passaged through two cell types induced more variable expression of CXCR3 isoforms. While infection with ZIKV-FLA/2 showed similar expression patterns to that of FLA/1, stromal MSCs infected with ZIKV-FLR/2 demonstrated variability in isoform expression with no significant differences. These data suggest that both isoforms CXCR3-A and CXCR3-B are upregulated during ZIKV infection of human prostate cells in a passage-dependent manner.

### CXCR3 signaling abrogates ZIKV replication in prostate cells in a passage-dependent manner

**Fig 8:**
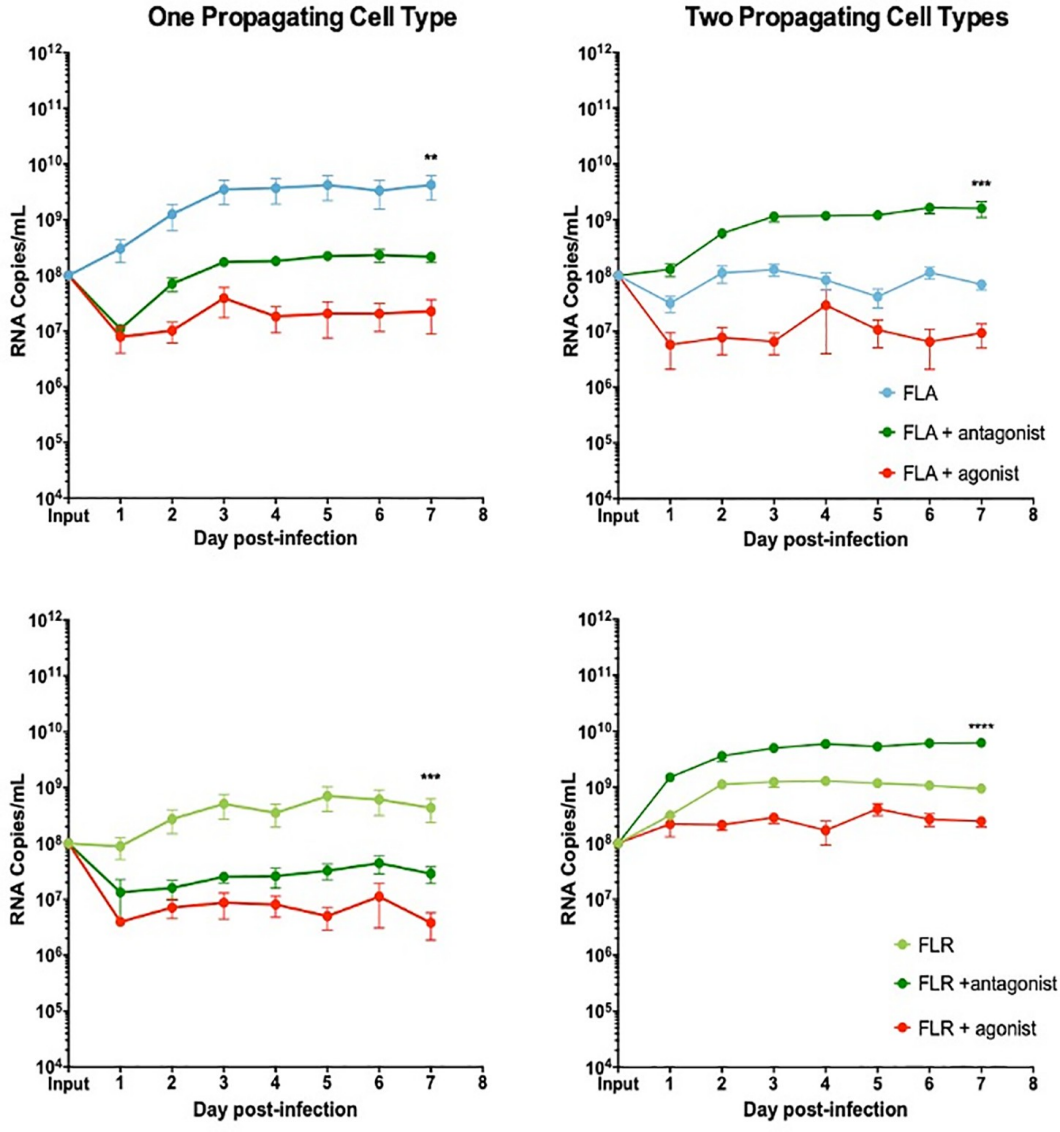
CXCR3 agonist and antagonist treatments alter ZIKV replication in prostate epithelial cell infection. PNT1a cells were treated with 3.75 ng/mL CXCR3-specific agonist (PS372424) or CXCR3-specific antagonist ((+/-) NBI-74330) for 3 hours prior to infection. Cells were infected with ZIKV-FLA or FLR propagated in only one cell type (left column), or after passage through two cell types (right column) at an MOI of 0.1. Infected cell supernatants were collected and RNA extracted daily up to 7 dpi. One-step qRT-PCR was performed with ZIKV specific primer and probe to assess viral replication. Data are from 2 or 3 independent experiments with 3 technical replicates each. Error bars are SEM. Statistical significance was determined using the D'Agostino & Pearson omnibus normality test, and a Friedman Test with Dunn’s multiple comparisons. Significance was ** = *p*<0.01; *** = *p*<0.005; **** = *p*<0.001.

To determine if the anti-viral effect of IP-10 was driven by CXCR3 signaling, prostate cells were treated with a CXCR3 specific agonist or antagonist 3 hours prior to ZIKV infection. To directly compare these data with the IP-10 treatment results, we pre-treated cells with the same amount of agonist or antagonist (3.5 ng/mL) for consistency. Pre-treatment with CXCR3 agonist reduced viral RNA by approximately 2 logarithms in PNT1a epithelial cells infected with ZIKV isolates propagated in a single cell type (**Fig 8**). Treatment with CXCR3 antagonist inhibited ZIKV replication by approximately 0.5–1 logarithms in PNT1a epithelial cells infected with ZIKV-FLA or FLR propagated in one cell type. PNT1a epithelial cells treated with CXCR3 agonist and infected with ZIKV propagated through a second cell type decreased ZIKV replication by approximately 1 logarithm. Conversely, treatment of epithelial cells with CXCR3 antagonist enhanced ZIKV replication by 1 logarithm above that of untreated cells when infected with isolates propagated from two cell types. These data indicate modulation of CXCR3 signaling significantly affects ZIKV viral RNA output.

Additionally, to evaluate the effects of CXCR3 modulation on infectious virus production, we performed limiting dilution assays. Infectious ZIKV production was assessed for ZIKV-FLA or FLA propagated through one or two cell types with CXCR3 agonist treatment. There were no significant changes in infectious ZIKV production in PNT1a cells treated with CXCR3 agonist, as compared to untreated or IP-10 treated cells (**Table 2**). Although ZIKV-FLR propagated through a second cell type produced greater amounts of virus, it was not considered statistically significant.

**Fig 9:**
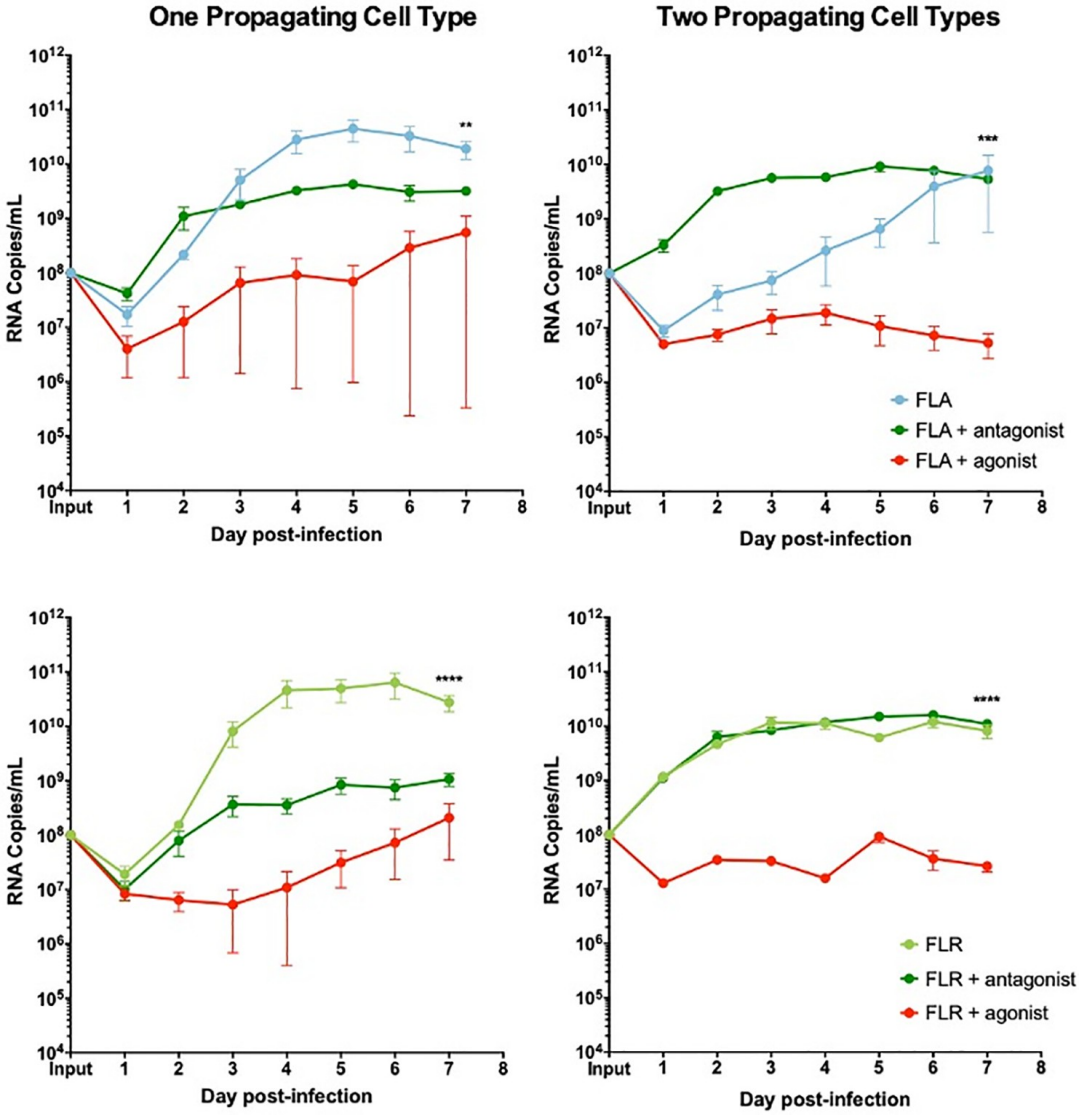
CXCR3 agonist and antagonist treatments alter ZIKV replication in prostate stromal MSC infection. MSCs were treated with 3.75 ng/mL CXCR3-specific agonist (PS372424) or CXCR3-specific antagonist ((+/-) NBI-74330) for 3 hours prior to infection. Cells were infected with ZIKV FLA or FLR propagated in only one cell type (left column), or after passage through the reciprocal cell types (right column) at an MOI of 0.1. Infected cell supernatants were collected and RNA extracted daily up to 7 dpi. One-step qRT-PCR was performed with ZIKV specific primer and probe to assess viral replication. Data are from 2 or 3 independent experiments with 3 technical replicates each. Error bars are SEM. Statistical significance was determined using the D'Agostino & Pearson omnibus normality test, and a Friedman Test with Dunn’s multiple comparisons. Significance was ** = *p*<0.01; *** = *p*<0.005; **** = *p*<0.001.

We demonstrated the same anti-viral role for CXCR3 signaling in ZIKV replication in prostate stromal MSCs. After pre-treating stromal MSCs with CXCR3 agonist and infecting with ZIKV propagated in one cell type, we saw a replication decrease of 2–3 logarithms (**Fig 9**). Conversely, pre-treatment of stromal MSCs with CXCR3 antagonist only partially restored ZIKV replication by increasing RNA levels by approximately 1 logarithm. Furthermore, the same trend was seen when stromal MSCs were infected with virus propagated through two cell types. CXCR3 agonist pre-treatment significantly abolished ZIKV replication by approximately 3 logarithms. However, CXCR3 antagonist pre-treatment was able to fully restore ZIKV replication to equal or higher levels than that of untreated and infected cells. From these data, we concluded that probing CXCR3 downstream signaling modulates ZIKV RNA output, and is significantly altered based on ZIKV isolate passage history.

To ensure ZIKV RNA replication differences were not due to changes in cellular viability resulting from IP-10, CXCR3 agonist, or CXCR3 antagonist treatments, we assessed viability of uninfected prostate cells with and without various pre-treatments by flow cytometry. At 7 days in culture, there were no significant differences in viability between untreated PNT1a epithelial cells and epithelial cells treated with IP-10 or CXCR3 antagonist (**S5 Fig**). However, there was a significant increase in cellular viability between untreated epithelial cells and epithelial cells treated with CXCR3 agonist. Conversely, there were no statistically significant changes between untreated stromal MSCs and IP-10, agonist, or antagonist treated MSCs. Taken together, these data indicate that pre-treatment with IP-10, CXCR3 agonist, or CXCR3 antagonist had no negative effects on overall cellular viability.

### CXCR3 signaling does not alter proliferation or viability of ZIKV infected cells

**Fig 10:**
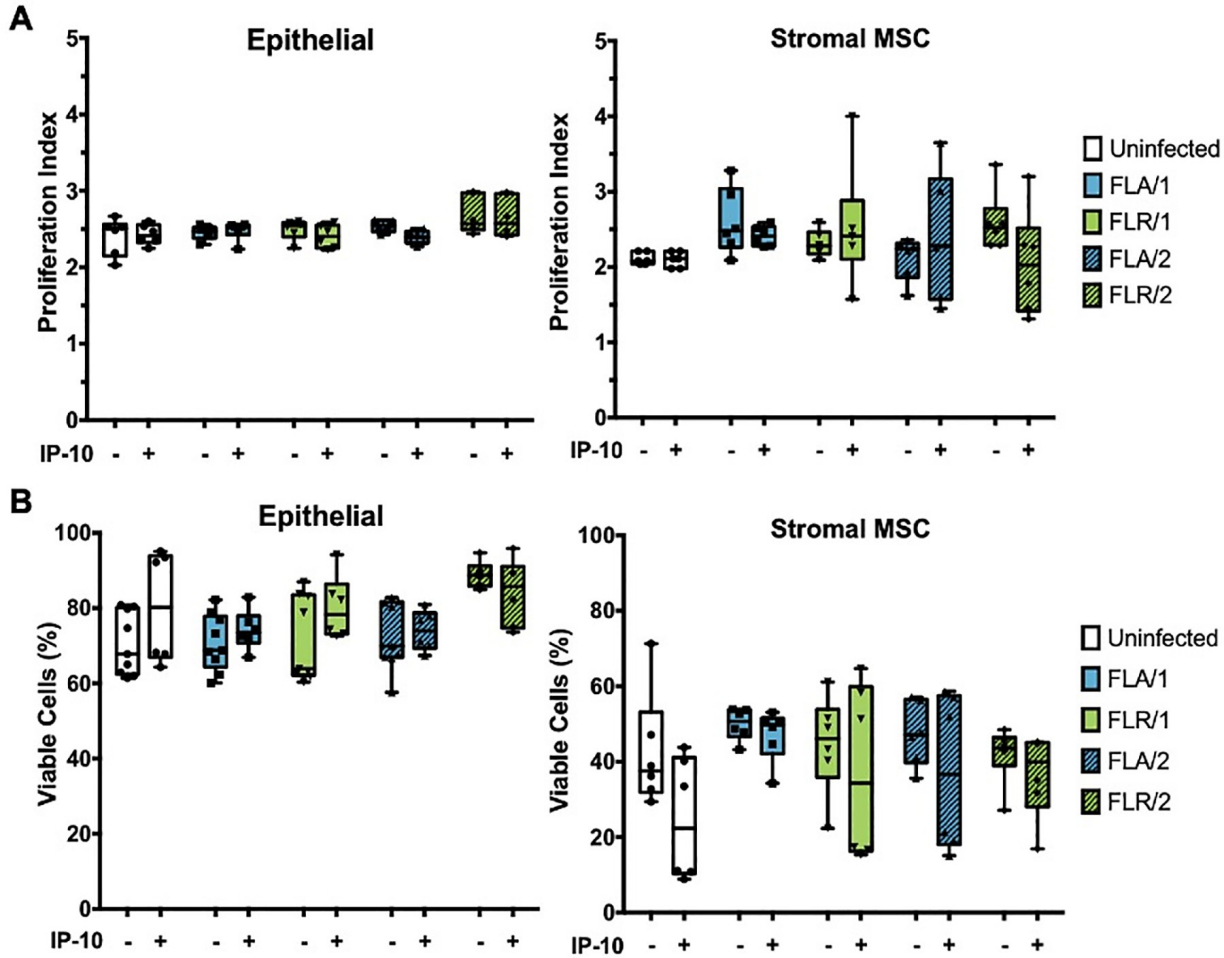
IP-10 treatment does not alter prostate cell proliferation or viability during ZIKV infection. Cells were stained with CellTrace Violet prior to infection with ZIKV isolates. Proliferation index indicates the cell divisions of PNT1a or MSCs after 7 dpi (**A**). Cells were infected with ZIKV and assessed for viability by flow cytometry at 7 dpi (**B**). Number of cell types used to propagate virus are indicated numerically after ZIKV isolate names. Data are presented as averages from 2 independent experiments with 3 technical replicates each. Error bars are SEM. Statistical significance was determined using one-way ANOVA followed by multiple comparison t-tests using Tukey correction. ns = not significant.

To understand the mechanism behind CXCR3 signaling anti-ZIKV effects, we investigated the main downstream outputs of CXCR3 signaling, cellular proliferation and survival [46]. To assess proliferative ability of ZIKV-infected prostate cells with or without exogenous IP-10, prostate cells were stained with a proliferation dye prior to pre-treatment and infection. Prostate epithelial cells and stromal MSCs were assessed for proliferative index by flow cytometry at 7 dpi. No significant changes in proliferation index were detected between infected and uninfected epithelial cells, as well as IP-10 treated or untreated epithelial cells (**Fig 10A**). Similarly, no significant differences were discernable in proliferation index between infected and uninfected, or IP-10 treated and untreated stromal MSCs (**Fig 10A**). These results were also observed for epithelial cells and stromal MSCs during ZIKV infection with isolates propagated in two cell types. Although there were higher levels of variation and an overall decreasing trend in proliferation index detected in stromal MSCs, none of the comparisons were statistically significant.

To determine if CXCR3 anti-ZIKV activity is a result of altered cellular viability during infection, we performed similar studies with pre-treatment of IP-10 and ZIKV infection in prostate cells. At 7 dpi, cells were stained with GhostDye and viability assessed by flow cytometry. There were no significant changes in viability between IP-10 treated, untreated, infected, and uninfected PNT1a epithelial cells (**Fig 10B**). Furthermore, there were no significant differences between cellular viability in stromal MSCs during IP-10 treatment or ZIKV infection, as compared to untreated or uninfected controls (**Fig 10B**). From these data, we can conclude that CXCR3 anti-ZIKV activity is not due to changes in cellular viability during infection or IP-10 stimulation.

### CXCR3 signaling induces limited apoptosis in ZIKV infected prostate cells

**Fig 11:**
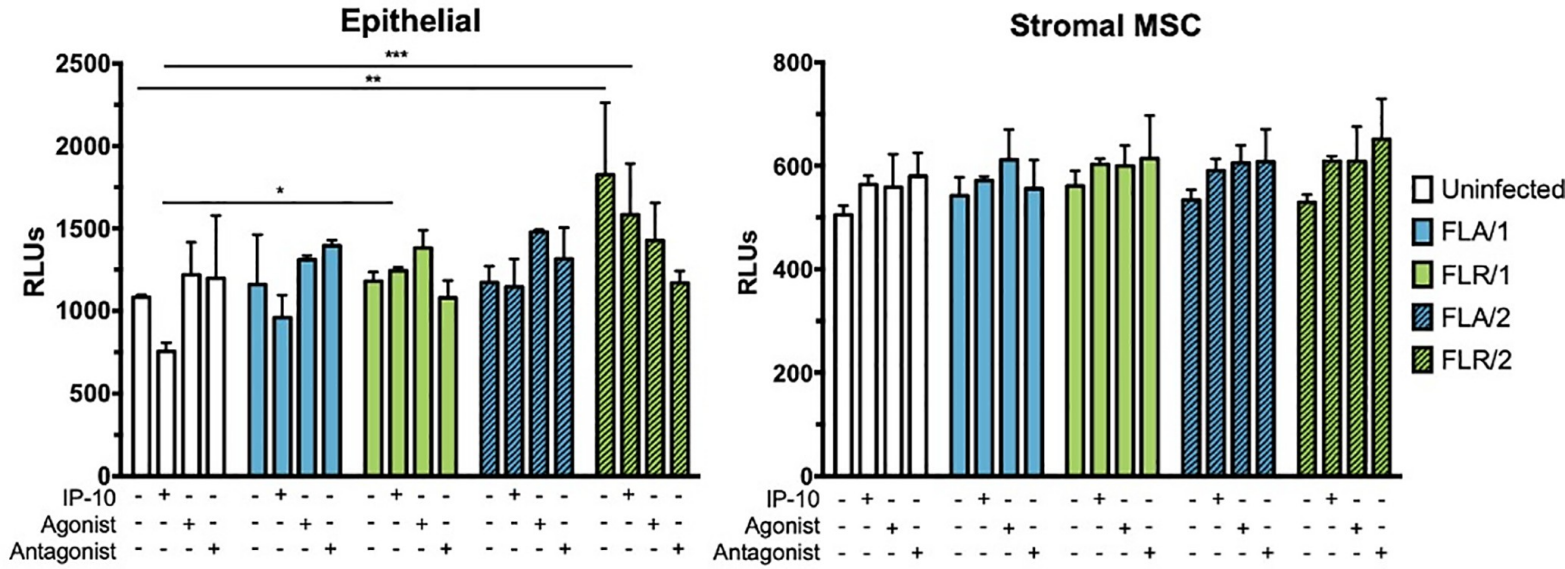
IP-10, CXCR3 agonist, or CXCR3 antagonist do not alter caspase activation during ZIKV prostate infection. Prostate epithelial cells and stromal MSCs were pretreated with IP-10, CXCR3 agonist, or CXCR3 antagonist 3 hours before infection. Cells were infected with ZIKV at an MOI of 0.1 and caspase induction measured at 24 hours post-infection by CaspaseGlo3/7 luminescence assay. Number of cell types used to propagate virus are indicated numerically after ZIKV isolate names. Data are from 2 independent experiments with 2 technical replicates each. Error bars are SEM. Statistical significance was determined using two-way ANOVA followed by multiple comparison t-tests using the Holm-Sidak correction. Significance was * = *p*<0.05; ** = *p*<0.01; *** = *p*<0.005; **** = *p*<0.001.

Although ZIKV infection or IP-10 treatment did not significantly alter viability of infected prostate cells at 7 dpi, we hypothesized that changes in viability could be occurring at an earlier time point during infection due to changes in signaling. Therefore, we investigated initial responses to infection by assaying early infection apoptosis induction. To probe apoptosis induction, we measured activation of caspases 3 and 7 at 24 hours-post infection, with and without IP-10, CXCR3 agonist, or CXCR3 antagonist pre-treatment. Caspase induction was unchanged in PNT1a epithelial cells during treatments and infection with ZIKV-FLA (**Fig 11**). However, there was a significant increase in caspase activity in untreated and IP-10 treated ZIKV-FLR-infected cells, regardless of virus passage history. ZIKV infection nor pre-treatments altered caspase induction levels in prostate stromal MSCs (**Fig 11**). Although IP-10, CXCR3 agonist, and CXCR3 antagonist treatments resulted in an increased trend of caspase activation of stromal MSCs, the data were not statistically different.

## Discussion

The sexual transmission of Zika virus makes it unique among the mosquito-borne viruses of humans. We have described the importance of the prostate in providing a site for long-term replication of this virus, and as a conduit for further human-to-human spread beyond the typical mosquito-bite transmission. Here we have shown that ZIKV and DENV-2 infections in prostate cells elicit isolate-specific cytokine profiles, including the production of IFNγ and IP-10. Furthermore, we demonstrated that IP-10 significantly inhibits ZIKV replication in human prostate cells and is controlled by CXCR3 chemokine signaling. CXCR3 isoforms are upregulated during prostate cell infection, and ZIKV replication differences are not due to changes in cellular proliferation, viability, or caspase induction. Overall, we have demonstrated an anti-viral role for IP-10/CXCR3 signaling during ZIKV infection of human prostate cells and that anti-viral activity is dampened by passaging virus through multiple cell types.

### Distinct cytokine profiles of ZIKV isolates

Cytokine profiling of ZIKV infections of prostate cells indicated clear differences in cytokine production based on ZIKV isolate history (**S2–S4 Figs**). ZIKV isolates FLA and FLR induced more robust cytokine profiles than that of ZIKV-HN16 or DENV-2. These data correspond to the lower viral RNA output in HN16 and lowest DENV-2 RNA in infected PNT1a cells. This could reflect Vero cell passaging of HN16, or the characteristic of DENV as a non-sexually transmitted *Flavivirus*, as it does not elicit the same cytokine responses as shown here. By isolating ZIKV directly from serum in different host cell types, different evolutionary pressures on the viruses may influence genetic variability. This genetic variability has been demonstrated by deep sequencing and sequence comparisons between human-derived and mosquito-derived ZIKV isolates [48]. ZIKV isolates derived from mosquitoes had significantly higher percentages of single nucleotide variants in genomic regions encoding the envelope, NS1, NS2A, NS3, and NS5 proteins, as compared to human-derived ZIKV sequences [48]. Differences in phenotypes have been shown in ZIKV isolated from different sources (macaque, human, or mosquito) and with variable passage histories (<10 to >100 passages) [49]. Changes that resulted from ZIKV genetic variability included virus infectivity, host cell viability, and viral growth kinetics in Vero cells and C6/36 mosquito cells [49]. However, our three strains of ZIKV are genetically similar [35, 36], isolated from the same Americas outbreak, and only differ by 7 amino acids (**Fig 1**). Furthermore, we were not able to find any of these nucleotide/amino acid differences associated with specific phenotype changes described in previous reports [24].

An alternative hypothesis addressing differences in cytokine production in ZIKV isolates could be due to post-translational modifications on different virus isolates, such as variable glycosylation moieties, based on their differences in isolation history. It is postulated that the two primary functions of glycosylation differences on viral glycoproteins are to protect the virus from the host immune system, and to facilitate viral entry into host cells [27]. Glycosylation of ZIKV virion envelope proteins have been shown to have an effect on viral infectivity, formation, and release of new virions in mammalian cell culture [28], as well as overall pathogenesis in mice models [50]. The N154Q mutation attenuating ZIKV pathogenesis and ablating ZIKV envelope glycosylation is not present in any of our ZIKV clinical isolates [50]. It has also been show that many Latin American ZIKV isolates have the most amino acid variability in the envelope and prM proteins, where glycosylation is expected to occur [51]. Future studies will determine glycosylation profiles of different ZIKV isolates, as well as conduct sequencing comparisons across isolates after passaging.

### Induction of IP-10 and ZIKV antiviral response

Additionally, two cytokines that were upregulated during ZIKV prostate cell infection were IFNγ and IP-10. While IFNγ is traditionally thought to be produced only by immune cells, our results add to the repertoire of cell types able to produce and respond to IFNγ, as there are no immune cells present in our culture system. Production of IFNγ was also reported in prostate basal epithelial cells [52], further supporting our findings. IP-10 is canonically known as an IFNγ-induced ISG, responsible for chemoattraction and adherence of immune cells [46, 53]. IP-10 has been associated with promoting (HIV, Herpes Simplex 2) or protecting against (Coronaviruses, Epstein Barr Virus) viral infection in different cell types [46, 54–59]. Additionally, IP-10 is associated with severity of infection caused by sexually-transmitted viruses, including HIV, HCV, and Herpes Simplex 2 [54, 58–60]. Furthermore, IP-10 can be upregulated in an IFN-independent manner through IRF3 and RIG-I-like receptor (RLR) signaling mechanisms during Hepatitis A virus infection [61]. Therefore, it is possible that IP-10 induction in prostate cells may be mediated by RLR detection of exogenous ZIKV RNAs with or without IFNs present [61].

Studies of ZIKV-infected patients have indicated higher expression of IP-10 and IFNγ in peripheral blood during acute infection, while serum levels of IFNγ were reduced during the convalescent phase [62–64]. Furthermore, ZIKV-infected patients showing neurological complications had higher expressions levels of IFNγ and IP-10, as compared to infected patients without neurological disease [63, 64]. An increase in IP-10 has been seen during *in vitro* ZIKV infections of human astrocytes and primary human testicular cells [65, 66]. Transcriptomic analysis of Sertoli cells infected with ZIKV have yielded confounding data. While one study has shown increases in IP-10 transcripts at 2 dpi, another shows a general increase in IFN-related transcripts but not specifically IP-10 [67, 68]. Additionally, elevated IP-10 levels have been described in the semen, blood plasma, and serum of ZIKV-infected patients [63, 69, 70]. Previous studies of ZIKV infection of human cells have also demonstrated an induction of IP-10 during *in vitro* infection of human neuroprogenitor stem cells [71]. Infection of mice via intravaginal inoculation induced high levels of IP-10 expression in neural stem cells, further elucidating a role for IP-10 in sexually-transmitted ZIKV infections [72]. However, the direct role of IP-10 in ZIKV urogenital tract infection has yet to be studied. In addition, other studies in human macrophages have described the ability of ZIKV to upregulate pro-inflammatory cytokines that are responsible for induction of anti-viral proteins, such as CH25H [73]. However, one limitation of this study includes using PNT1a cells for assessing cytokine production. PNT1a cells were immortalized by SV40 Large T antigen, which is known to upregulate transcription of ISGs and IFNs [74]. It is possible that Large T antigen had variable effects on cytokine production, as well as the inhibitory effects of IP-10 on ZIKV replication. Future studies will include non-transformed primary prostate epithelial cells to ensure cytokine profiles are unaltered by Large T antigen.

Our results have shown that treatment with IP-10 before or after ZIKV infection significantly reduced viral replication in human prostate cells, indicating the therapeutic potential of this chemokine/cytokine. Harnessing cytokine signaling pathways has been proposed to treat other sexually-transmitted virus infections, including IL-7 in HIV, IFNα in HCV, and JAK/STAT pathways in other flavivirus infections [75–79]. These studies demonstrated that the use of cytokine treatment in infected human patients could reduce viral replication when used at relatively high concentrations (500 units/10 μg per kg) over long time courses (three times weekly for two to 28 weeks) [78, 79]. Conversely, our studies used IP-10 at low (ng) concentrations for a single treatment prior to infection and culturing cells up to 7 dpi. These results indicate that ZIKV replication is very sensitive to IP-10/CXCR3 signaling. Additional studies will assess the efficacy of IP-10 treatment on long-term ZIKV infections and gauge if additional treatments are necessary to abrogate viral replication.

### Phenotypic effects on viral passaging

Virus propagated through two cell types showed significant loss of anti-viral ability with IP-10 treatment. Our results indicate that virus passage level has a major effect on anti-viral sensitivity to IP-10, and this results in significant phenotypic changes. This is consistent with previous reports describing decreased sensitivity to IFN-induced anti-viral responses in the highly-passaged African strain MR-766 (Uganda), as compared to lower-passage Asian isolates [80]. Thus, cell culture passage of ZIKV can lead to significant differences in results, and others have demonstrated this in cell culture by enrichment of ZIKV phenotypic variants [24, 80]. The 2015 ZIKV isolate from Puerto Rico, PRVABC59, was passaged three times in Vero cells, and resulted in non-synonymous mutations (V330L/W98G) in the envelope and NS1 proteins [24]. These variants were enriched in cell culture passaging and demonstrated decreased pathogenesis, attenuated virulence, and decreased dissemination in mice based on their survival, weight change, and viremia, respectively [24].

In our studies, pre-treatment with CXCR3 agonist significantly inhibited viral replication, indicating that stimulating CXCR3 signaling has a direct anti-viral effect.

When prostate cells were pre-treated with CXCR3 antagonist, we observed either a partial rescue or enhancement of viral replication, based on the number of cell types the virus had been propagated in. Based on previous data, we expected a boost in viral replication with ZIKV propagated through a single cell type after CXCR3 antagonist treatment, and an altered phenotype after ZIKV passaging. However, the enhancement of viral replication was only achieved with virus propagated through two cell types. Although these data contradicted our expected results, our proposed model of altered viral phenotypes after additional cell culture passaging holds true. Future studies addressing the mechanism behind CXCR3-induced anti-viral response may shed light on this unexpected result. Furthermore, this altered phenotype was also prostate cell type-dependent and was more robust in stromal MSCs than epithelial cells. These differences may be due to increased ZIKV susceptibility in stem cells [81–83].

Overall, our results concur with previously published studies that described different phenotypes across strains and after passaging [84, 85]. High passage viral strains, including PRVABC59 (Puerto Rico) and MR-766 (Uganda), grow to much higher levels in cell culture compared to clinical ZIKV isolates, and are therefore not representative of clinical ZIKV infection [25, 84, 85]. This is also demonstrated by differences of IP-10 induction magnitude among strains, since a clinical ZIKV isolate from Brazil (PE243) induced significantly lower concentrations of IP-10 than a Cambodian, cell culture-adapted, high passaged strain (FSS13025) during *in vitro* infections [71]. Other related flaviviruses, such as WNV, have also shown differential induction of IP-10 based on virus passaged through mosquito or mammalian cells [86]. IP-10 was significantly higher in human plasmacytoid dendritic cells infected with WNV derived from mammalian cells, while there was no induction of IP-10 during infection with WNV propagated in mosquito cells [86]. Taken together, our data show that passaging cell type has a profound effect on the virus phenotype we were testing, in this case, the anti-viral effects of IP-10/CXCR3 signaling.

### CXCR signaling and potential antiviral mechanisms

ZIKV infection of prostate cells induced a significant upregulation of multiple CXCR3 isoforms, including primary isoforms CXCR3-A and CXCR3-B, as demonstrated by qRT-PCR [87]. This is a novel finding, as other studies have not previously shown CXCR3-specific upregulation by transcriptomic analysis [67, 68]. However, as mRNA levels may not correspond to increased protein levels, additional experiments are needed to directly assess if changes in isoforms reflect changes in CXCR3 protein. Nevertheless, isoform induction varied by prostate cell type, ZIKV isolate, and number of cell passages. Although an upregulation of both primary isoforms was shown here, a less well-characterized isoform, CXCR3-alt, can be produced by post-transcriptional exon skipping in human peripheral blood mononuclear cells [87, 88]. Thus, it would be important to study differences in infectivity between CXCR3-A and -B expressing cells, as well as the role of truncated isoform CXCR3-alt in ZIKV infection of prostate cells.

Although higher levels of CXCR3 were detected during infection, we identified no significant differences in cellular proliferation or viability. Previous studies of ZIKV-infection of neural progenitor cells *in vitro* and *in vivo* showed decreased proliferation [81, 89]. However, these studies were conducted with a prototype, high passaged ZIKV strain, MR-766 (Uganda), and in immunocompromised (IRF^-/-^) mice [81, 89]. It is well documented that IFN responses are critical in promoting or protecting against ZIKV infections [90–92]. IFNα and IFNβ are selectively inhibited during infection by ZIKV by downstream degradation of IFN signaling molecule, STAT2 [90, 91]. Conversely, data have also shown that IFNγ enhances viral replication in placental and glioblastoma cells [92]. Therefore, studies with IFN-deficient models would not reflect clinical infections of humans [93]. Also, we showed significantly higher induction of caspases by virus isolate ZIKV-FLR, regardless of passage history. Previous studies of ZIKV infections have demonstrated induction of apoptosis in HUVEC cells when infected at a higher MOI of 5 [94]. Although it reflects more biologically relevant conditions, it is possible that the MOI used in our studies was too low to distinguish differences in viability by flow cytometry. Additional studies utilizing live cell fluorescent imaging for caspase activation, and MTT activation for proliferative ability would add insight to these results.

It is possible that the anti-viral role of CXCR3 could be mediated by multiple mechanisms, including host cell entry, viral RNA production, or chemokine signaling. CXCR3 is a G-protein-coupled receptor (GPCR) associated with controlling downstream cell growth and survival [87]. Ligand-GPCR interactions ultimately result in ligand-induced receptor activation and internalization of membrane-bound GPCRs [87]. Viral hijacking of GPCRs has been shown to occur in several viral diseases. HIV uses the GPCR, CXCR4, as an essential cofactor for viral entry into host cells [95, 96]. However, the mechanisms of action for CXCR3 in ZIKV infection have not been elucidated, and here we report the first studies indicating its anti-viral role. While there are several elucidated primary receptors responsible for flavivirus binding and entry into host cells, there are many speculated low-affinity co-receptors as well. Future studies will investigate whether CXCR3 acts as a viral cofactor for ZIKV entry, and this could lead to testing specific inhibitors for prostate infections. Additionally, it is possible that CXCR3 signaling may induce other factors responsible for inhibiting RNA replication. Other CXCR3-associated factors could mediate infection inhibition by directly influencing RNA synthesis or indirectly priming uninfected cells to decrease spread of infection. Based on results from IP-10, agonist, and antagonist treatment experiments, we have shown that receptor internalization and downstream CXCR3 signaling are necessary for full CXCR3-mediated anti-viral effects. Future mechanistic studies will address which CXCR3 signaling factors could be playing a role and how they are facilitating these anti-viral effects.

Taken together, our data indicate that IP-10/CXCR3 signaling restrict ZIKV RNA production in human prostate cells, without negatively affecting proliferation or viability. However, we understand that there are some limitations of interpreting the effects of IP-10 on overall ZIKV infection. Due to various constraints, we were unable to perform infectious virus titrations on all samples during stromal MSC ZIKV infection; we therefore extrapolated from the titration experiments in PNT1a cells, by limiting dilution assays. While these limitations dampen the overall interpretation of IP-10/CXCR3 effects on ZIKV replication, it is an important starting point for investigating IP-10/CXCR3 effects during ZIKV infection. By presenting early data here, supporting an anti-viral role for IP-10/CXCR3, additional studies can fully address the mechanism behind ZIKV RNA reduction by IP-10/CXCR3 modulation.

Importantly, ZIKV replication did not seem to affect prostate cell viability or apoptosis, suggesting that chronicity is possible in this organ. Future studies also need to address the mechanism of IP-10/CXCR3 anti-viral responses in ZIKV infections of human testicular and epididymal cells. As the testes are another suspected site of ZIKV persistence, these studies could highlight similarities between chronically ZIKV-infected urogenital tract tissues. If IP-10/CXCR3 restricts ZIKV infection in other urogenital tract tissues and the mechanism of action is identified, IP-10/CXCR3 signaling could serve as a potential therapeutic target to ultimately interrupt sexually-transmitted ZIKV infections. Several CXCR3-based therapies have been developed to either dampen inflammatory signals induced by CXCR3 ligands, or prevent CXCR3 activation [97]. Human monoclonal antibodies against IP-10 (MDX-1100/BMS-936557 and NI-0801) have been well tolerated in clinical trials to treat several inflammatory conditions, such as rheumatoid arthritis, cirrhosis, and inflammatory bowel disorder. Furthermore, CXCR3 ligand levels can act as prognostic or diagnostic biomarkers for infection. IP-10 levels are currently being correlated with disease severity in HCV infection, and are used as markers for how well patients will respond to multiple HCV therapies [97]. Based on our results, we postulate that CXCR3-induced autocrine or paracrine signaling may be responsible for stopping the spread of virus from infected cells to uninfected neighboring cells. Therefore, CXCR3-ligand based therapies could be useful in stopping ZIKV urogenital tract infections.

## Supporting information

S1 Fig ZIKV isolates have similar growth phenotypes in commonly used cell types, except FLA in Vero cells. Infected cell supernatants were collected and RNA extracted daily up to 7 dpi. One-step qRT-PCR was performed with ZIKV specific primer and probe to assess viral replication. Growth curves of ZIKV isolates FLA, FLR, and HN16 in C6/36 mosquito cells at MOI 1 and 0.1 (**A**), or Vero cells at an MOI of 1 and 0.1 (**B**). Data are from 2 independent experiments with 3 technical replicates each. Error bars are SEM. Statistical significance was determined using the D'Agostino & Pearson omnibus normality test, and a repeated measures two-way ANOVA with multiple comparison t-tests using Tukey correction. Significance was ns = not significant; **** = *p*<0.001.
(TIFF)

S2 Fig IFN/ISGs do not contribute to distinct cytokine profiles of ZIKV/DENV isolates in prostate epithelial cell (LNCaP) infection. Infected cell supernatants were collected at 1, 3, and 6 dpi and assessed for expression of 41 cytokines using MAGPIX multiplex immunoassay. (**A**) Heat map showing differences in cytokine production during ZIKV FLA, FLR, HN16, and DENV-2 infections. Data is shown as Log_10_ Fold Change of cytokine levels compared to uninfected controls. Red shading corresponds to cytokine upregulation, while blue shading corresponds to downregulation. Darker boxes indicate more marked expression changes. Data are from 2 independent experiments with 3 technical replicates each. (**B**) Principal component analysis depicts differential clustering of ZIKV FLA, FLR, HN16, and DENV-2 at all time points of infection, and each time point post-infection individually. Each dot represents a different time point and are labeled accordingly.
(TIFF)

S3 Fig ZIKV isolates and DENV elicit distinct cytokine profiles in prostate epithelial cell (PNT1a) infections. Infected cell supernatants were collected at 1, 3, and 6 dpi and assessed for expression of 41 cytokines using MAGPIX multiplex immunoassay. (**A**) Heat map showing differences in cytokine production during ZIKV FLA, FLR, HN16, and DENV-2 infections. Data is shown as Log_10_ Fold Change of cytokine levels compared to uninfected controls. Red shading corresponds to cytokine upregulation, while blue shading corresponds to downregulation. Darker boxes indicate more marked expression changes. Data are from 2 independent experiments with 3 technical replicates each. (**B**) Principal component analysis depicts differential clustering of ZIKV FLA, FLR, HN16, and DENV-2 at all time points of infection, and each time point post-infection individually. Each dot represents a different time point and are labeled accordingly.
(TIFF)

S4 Fig ZIKV isolates and DENV elicit distinct cytokine profiles in prostate Mesenchymal Stem Cell (MSC) infections. Infected cell supernatants were collected at 1, 3, and 6 dpi and assessed for expression of 41 cytokines using MAGPIX multiplex immunoassay. (**A**) Heat map showing differences in cytokine production during ZIKV FLA, FLR, HN16, and DENV-2 infections. Data is shown as Log_10_ Fold Change of cytokine levels compared to uninfected controls. Red shading corresponds to cytokine upregulation, while blue shading corresponds to downregulation. Darker boxes indicate more marked expression changes. Data are from 2 independent experiments with 3 technical replicates each. (**B**) Principal component analysis depicts differential clustering of ZIKV FLA, FLR, HN16, and DENV-2 at all time points of infection, and each time point post-infection individually. Each dot represents a different time point and are labeled accordingly.
(TIFF)

S5 Fig IP-10, CXCR3 agonist, or CXCR3 antagonist have no negative effects on viability of uninfected prostate cells. PNT1a epithelial cells or stromal MSCs were treated with 3.75 ng/mL CXCR3-specific agonist (PS372424) or CXCR3-specific antagonist ((+/-) NBI-74330) for 3 hours, approximately 24 hours after culturing. Cells were collected and stained with GhostDye (Tonbo) after 7 days of culturing, and assessed for viability by flow cytometry. Data are from 2 independent experiments with 3 technical replicates each. Error bars are SEM. Statistical significance was determined using an ordinary one-way ANOVA with multiple comparisons and Tukey’s correction. Significance was ns = not significant, ** = *p*<0.01.
(TIFF)

S1 Table Cytokines assessed via multiplex cytometric bead array assay. (DOCX)

S2 Table Primers and probes used to detect ZIKV RNA or DENV-2 RNA by qRT-PCR. (DOCX)

S1 Dataset Includes excel spreadsheets of all raw data accumulated from multiplex cytometric bead array assays to quantify cytokine concentrations. (XLSX)
