# Supplementary figures and images for: IP-10 and CXCR3 signaling inhibit Zika virus replication in human prostate cells
Source: PLoS One. 2020 Dec 30;15(12):e0244587. doi: 10.1371/journal.pone.0244587 (PMC7773246; doi:10.1371/journal.pone.0244587)

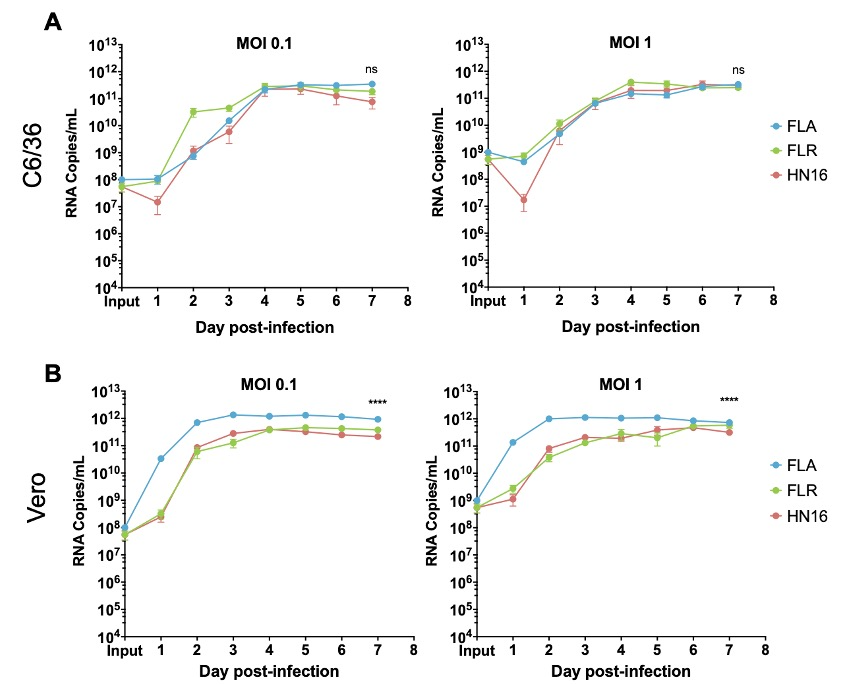

Supplement: S1 Fig — Infected cell supernatants were collected and RNA extracted daily up to 7 dpi. One-step qRT-PCR was performed with ZIKV specific primer and probe to assess viral replication. Growth curves of ZIKV isolates FLA, FLR, and HN16 in C6/36 mosquito cells at MOI 1 and 0.1 (A), or Vero cells at an MOI of 1 and 0.1 (B). Data are from 2 independent experiments with 3 technical replicates each. Error bars are SEM. Statistical significance was determined using the D'Agostino & Pearson omnibus normality test, and a repeated measures two-way ANOVA with multiple comparison t-tests using Tukey correction. Significance was ns = not significant; **** = p<0.001. (TIFF) [file pone.0244587.s001.tiff]

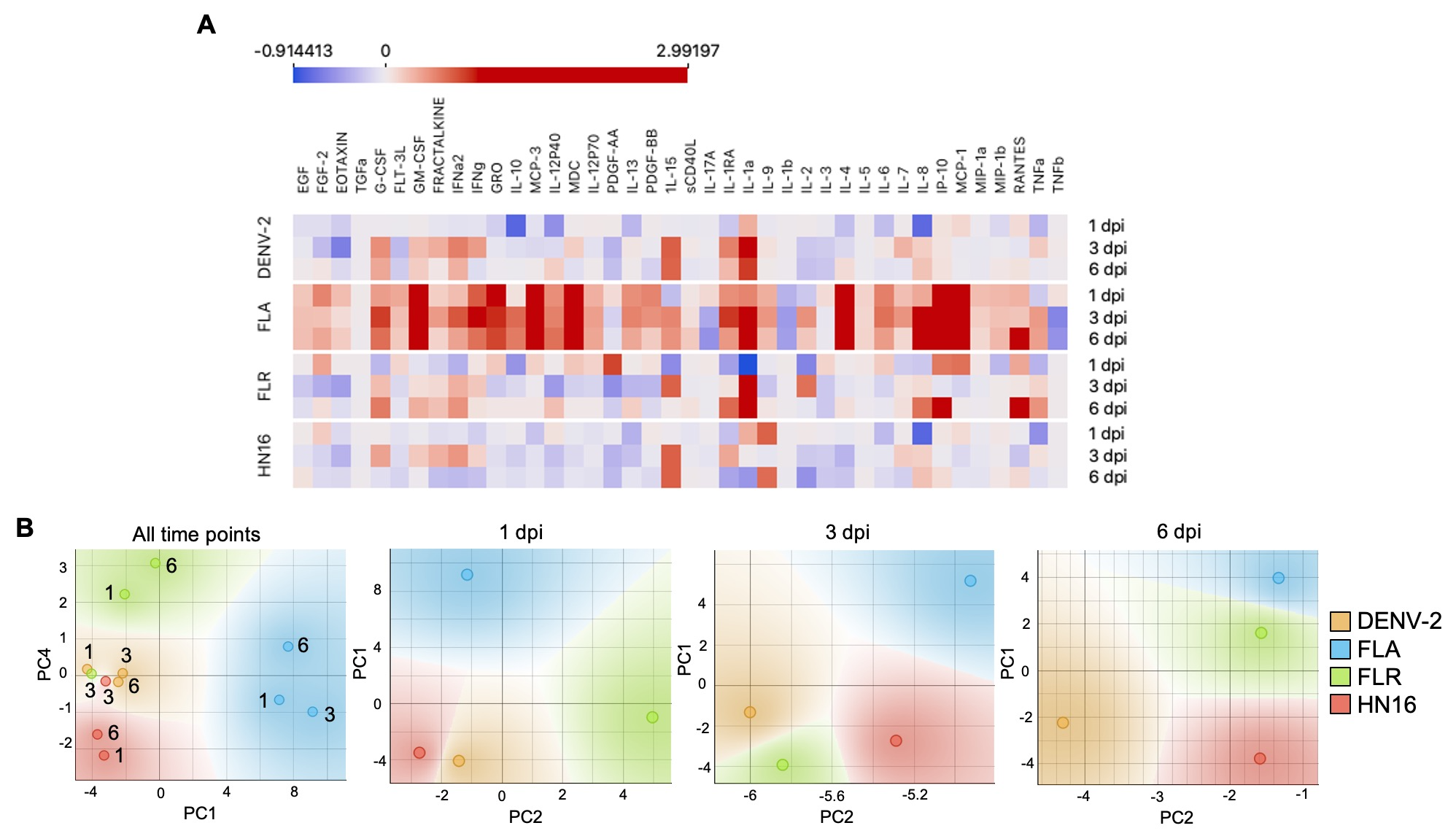

Supplement: S2 Fig — Infected cell supernatants were collected at 1, 3, and 6 dpi and assessed for expression of 41 cytokines using MAGPIX multiplex immunoassay. (A) Heat map showing differences in cytokine production during ZIKV FLA, FLR, HN16, and DENV-2 infections. Data is shown as Log10 Fold Change of cytokine levels compared to uninfected controls. Red shading corresponds to cytokine upregulation, while blue shading corresponds to downregulation. Darker boxes indicate more marked expression changes. Data are from 2 independent experiments with 3 technical replicates each. (B) Principal component analysis depicts differential clustering of ZIKV FLA, FLR, HN16, and DENV-2 at all time points of infection, and each time point post-infection individually. Each dot represents a different time point and are labeled accordingly. (TIFF) [file pone.0244587.s002.tiff]

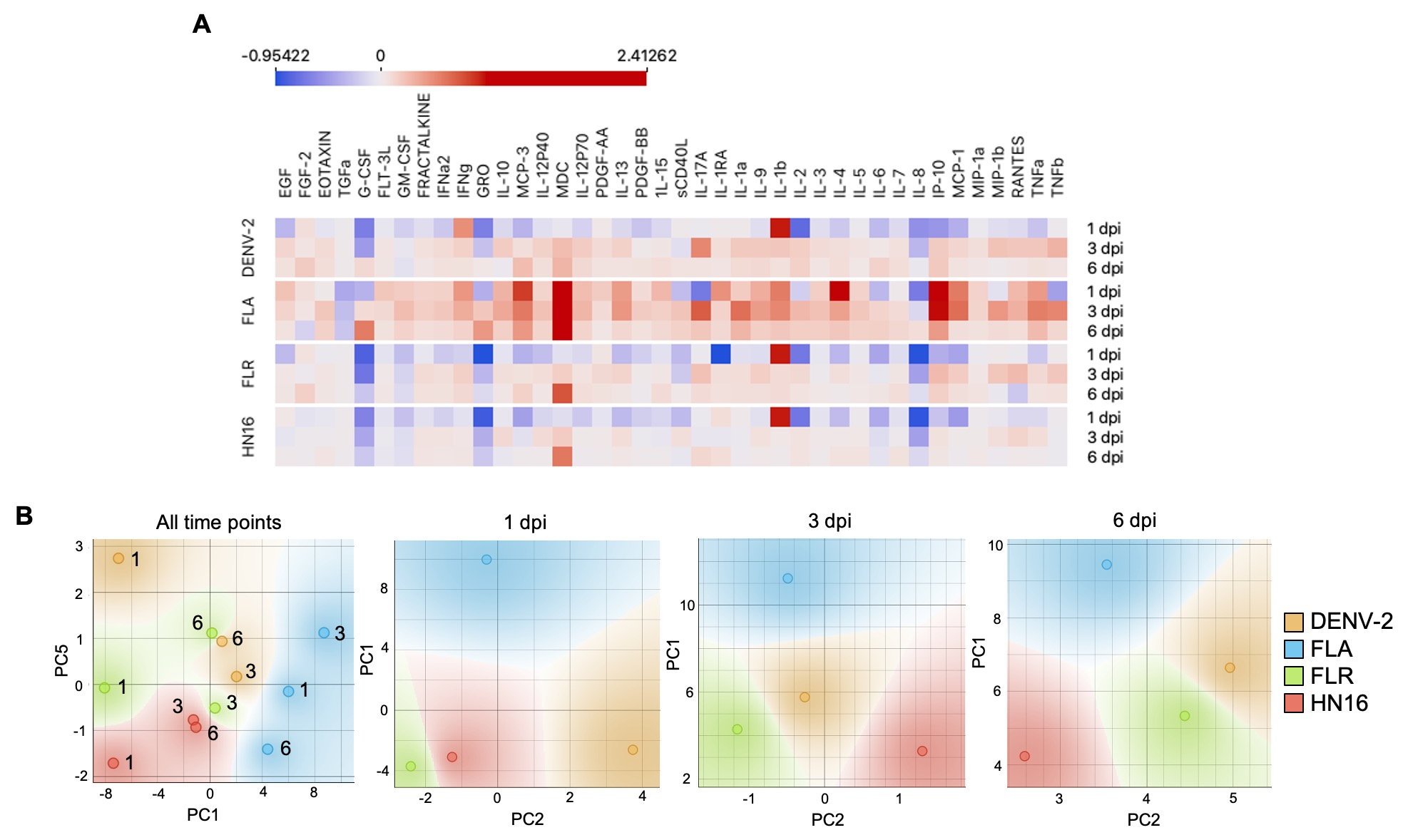

Supplement: S3 Fig — Infected cell supernatants were collected at 1, 3, and 6 dpi and assessed for expression of 41 cytokines using MAGPIX multiplex immunoassay. (A) Heat map showing differences in cytokine production during ZIKV FLA, FLR, HN16, and DENV-2 infections. Data is shown as Log10 Fold Change of cytokine levels compared to uninfected controls. Red shading corresponds to cytokine upregulation, while blue shading corresponds to downregulation. Darker boxes indicate more marked expression changes. Data are from 2 independent experiments with 3 technical replicates each. (B) Principal component analysis depicts differential clustering of ZIKV FLA, FLR, HN16, and DENV-2 at all time points of infection, and each time point post-infection individually. Each dot represents a different time point and are labeled accordingly. (TIFF) [file pone.0244587.s003.tiff]

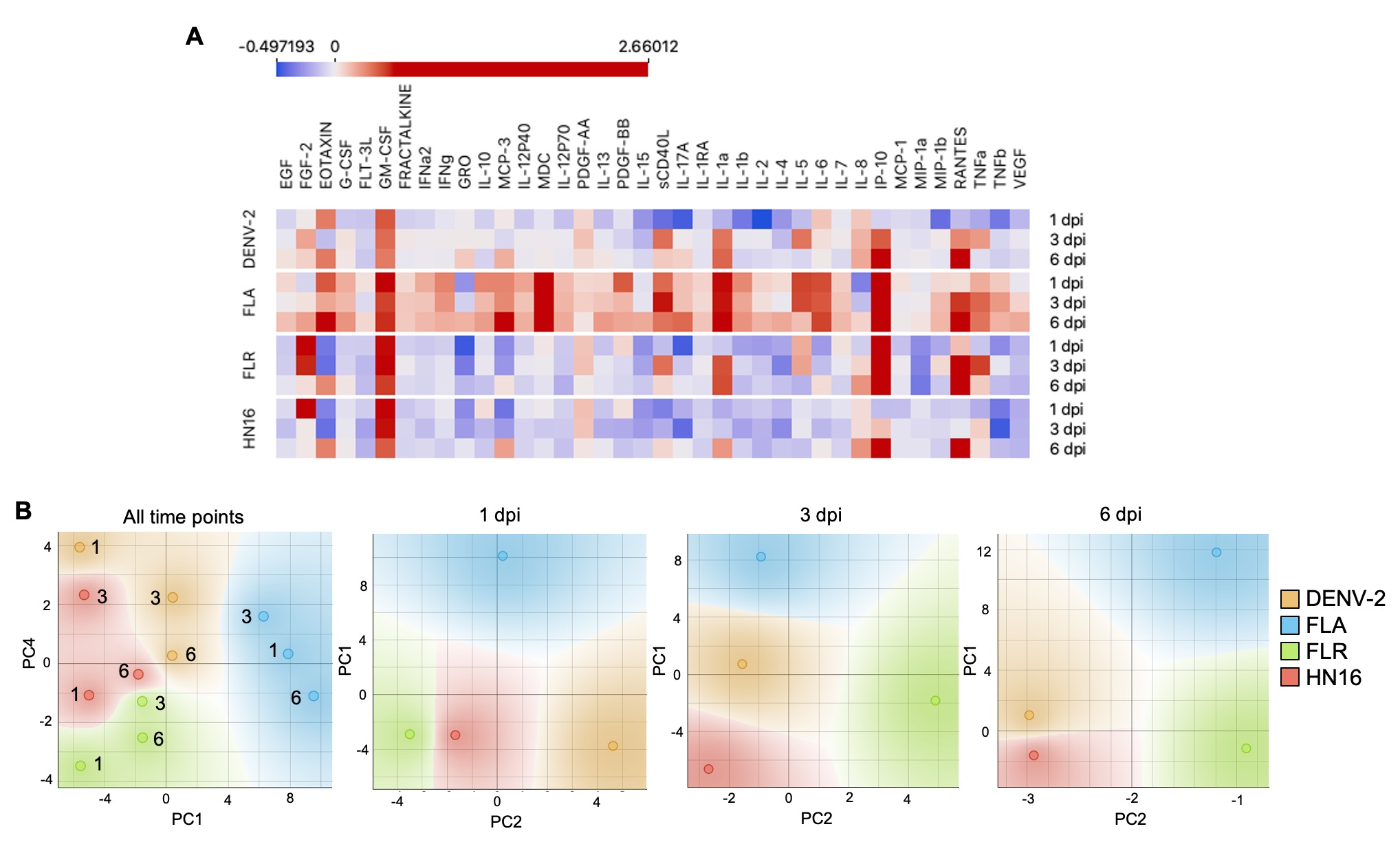

Supplement: S4 Fig — Infected cell supernatants were collected at 1, 3, and 6 dpi and assessed for expression of 41 cytokines using MAGPIX multiplex immunoassay. (A) Heat map showing differences in cytokine production during ZIKV FLA, FLR, HN16, and DENV-2 infections. Data is shown as Log10 Fold Change of cytokine levels compared to uninfected controls. Red shading corresponds to cytokine upregulation, while blue shading corresponds to downregulation. Darker boxes indicate more marked expression changes. Data are from 2 independent experiments with 3 technical replicates each. (B) Principal component analysis depicts differential clustering of ZIKV FLA, FLR, HN16, and DENV-2 at all time points of infection, and each time point post-infection individually. Each dot represents a different time point and are labeled accordingly. (TIFF) [file pone.0244587.s004.tiff]

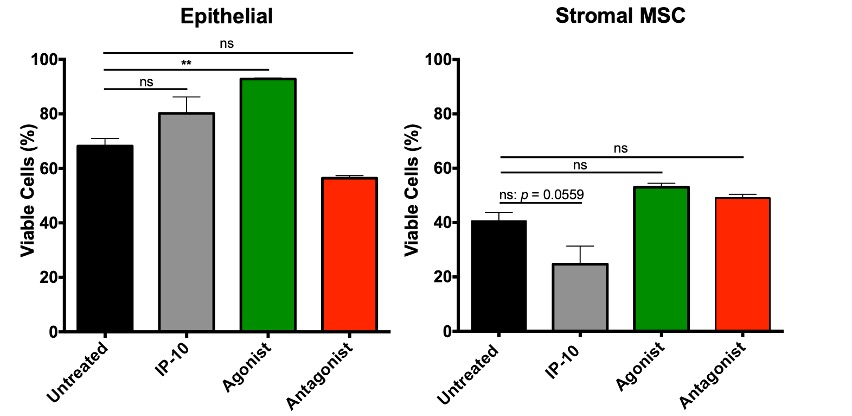

Supplement: S5 Fig — PNT1a epithelial cells or stromal MSCs were treated with 3.75 ng/mL CXCR3-specific agonist (PS372424) or CXCR3-specific antagonist ((+/-) NBI-74330) for 3 hours, approximately 24 hours after culturing. Cells were collected and stained with GhostDye (Tonbo) after 7 days of culturing, and assessed for viability by flow cytometry. Data are from 2 independent experiments with 3 technical replicates each. Error bars are SEM. Statistical significance was determined using an ordinary one-way ANOVA with multiple comparisons and Tukey’s correction. Significance was ns = not significant, ** = p<0.01. (TIFF) [file pone.0244587.s005.tiff]
