# Supplementary material for: IP-10 and CXCR3 signaling inhibit Zika virus replication in human prostate cells
Source: PLoS One. 2020 Dec 30;15(12):e0244587. doi: 10.1371/journal.pone.0244587 (PMC7773246; doi:10.1371/journal.pone.0244587)
Supplement: S1 Table — (DOCX) [file pone.0244587.s006.docx]

**S1 Table. Cytokines Assessed via Multiplex Cytometric Bead Array Assay.**

| **Cytokine** | **Functional Group(s)** | **Cytokine** | **Functional Group(s)** |
| --- | --- | --- | --- |
| EGF | Growth Factor | IL-12 (p40) | Pro-Inflammatory, T_H_1 |
| Eotaxin | Chemokine | IL-12 (p70) | Pro-Inflammatory, T_H_1 |
| FGF-2 | Growth Factor | IL-13 | T_H_2 |
| Flt-3L | Colony Stimulating Factor | IL-15 | T_H_1 |
| Fractalkine | Chemokine | IL-17A | Pro-Inflammatory |
| G-CSF | Colony Stimulating Factor | IP-10 | Chemokine |
| GM-CSF | Colony Stimulating Factor | MCP-1 | Chemokine |
| GRO | Chemokine | MCP-3 | Chemokine |
| IFNα | Pro-Inflammatory | MDC | Chemokine |
| IFNγ | Pro-Inflammatory, T_H_1 | MIP-1α | Chemokine |
| IL-1RA | Anti-Inflammatory | MIP-1β | Chemokine |
| IL-1α | Pro-Inflammatory | PDGF-AA | Growth Factor |
| IL-1β | Pro-Inflammatory | PDGF-BB | Growth Factor |
| IL-2 | T_H_1 | RANTES | Chemokine |
| IL-3 | Colony Stimulating Factor | sCD40L | Pro-Inflammatory |
| IL-4 | Anti-Inflammatory, T_H_2 | TGFα | Growth Factor |
| IL-5 | Anti-Inflammatory, T_H_2 | TNFα | Pro-Inflammatory, T_H_1 |
| IL-6 | Pro-Inflammatory, T_H_2 | TNFβ | Pro-Inflammatory, T_H­_1 |
| IL-7 | Colony Stimulating Factor | VEGF | Growth Factor |
| IL-8 | Chemokine |  |  |
| IL-9 | T_H_2 |  |  |
| IL-10 | Anti-Inflammatory, T_H_2 |  |  |

Cytokine names and functional groupings described for all 41 cytokines assessed by Milliplex.
