# Supplementary material for: IP-10 and CXCR3 signaling inhibit Zika virus replication in human prostate cells
Source: PLoS One. 2020 Dec 30;15(12):e0244587. doi: 10.1371/journal.pone.0244587 (PMC7773246; doi:10.1371/journal.pone.0244587)
Supplement: S2 Table — (DOCX) [file pone.0244587.s007.docx]

**S2 Table. Primers and probes used to detect ZIKV RNA or DENV-2 RNA by qRT-PCR.**

| **Virus** | **Primer/Probe** | **Sequence (5’-3’)** | **Genome Position** | **Reference** |
| --- | --- | --- | --- | --- |
| ZIKV | ZIKV 1086 | CCGCTGCCCAACACAAG | 1086-1102 | [41] |
|  | ZIKV 1107-FAM | AGCCTACCTTGACAAGCAGTCAGACACTCAA | 1107-1137 |  |
|  | ZIKV 1162c | CCACTAACGTTCTTTTGCAGACAT | 1162-1137 |  |
| DENV-2 | DENV2/141 | GCTGAAACGCGAGAGAAACC | 141-160 | [40, 42] |
|  | DENV2/177-FAM | AGCATTCCAAGTGAGAATCTCTTTGTCAGCTGT | 177-209 |  |
|  | DENV2/234 | CAGTTTTAITGGTCCTCGTCCCT | 212-234 |  |

Primer and probe nomenclature, sequences, position in the genome, and initial sequence references are included.
